# Mitochondrial DNA variants correlate with symptoms in myalgic encephalomyelitis/chronic fatigue syndrome

**DOI:** 10.1186/s12967-016-0771-6

**Published:** 2016-01-20

**Authors:** Paul Billing-Ross, Arnaud Germain, Kaixiong Ye, Alon Keinan, Zhenglong Gu, Maureen R. Hanson

**Affiliations:** Division of Nutritional Sciences, Cornell University, Ithaca, NY 14853 USA; Department of Molecular Biology and Genetics, Cornell University, Ithaca, NY 14853 USA; Department of Biological Statistics and Computational Biology, Cornell University, Ithaca, NY 14853 USA

**Keywords:** Myalgic encephalomyelitis (ME), Chronic fatigue syndrome (CFS), Next-generation sequencing, Mitochondrial DNA, mtDNA, Heteroplasmy, Association, SNPs, Haplogroup, Variants

## Abstract

**Background:**

Mitochondrial dysfunction has been hypothesized to occur in Myalgic Encephalomyelitis/Chronic Fatigue Syndrome (ME/CFS), a disease characterized by fatigue, cognitive difficulties, pain, malaise, and exercise intolerance. We investigated whether haplogroup, single nucleotide polymorphisms (SNPs), or heteroplasmy of mitochondrial DNA (mtDNA) were associated with health status and/or symptoms.

**Methods:**

Illumina sequencing of PCR-amplified mtDNA was performed to analyze sequence and extent of heteroplasmy of mtDNAs of 193 cases and 196 age- and gender-matched controls from DNA samples collected by the Chronic Fatigue Initiative. Association testing was carried out to examine possible correlations of mitochondrial sequences with case/control status and symptom constellation and severity as reported by subjects on Short Form-36 and DePaul Symptom Questionnaires.

**Results:**

No ME/CFS subject exhibited known disease-causing mtDNA mutations. Extent of heteroplasmy was low in all subjects. Although no association between mtDNA SNPs and ME/CFS vs. healthy status was observed, haplogroups J, U and H as well as eight SNPs in ME/CFS cases were significantly associated with individual symptoms, symptom clusters, or symptom severity.

**Conclusions:**

Analysis of mitochondrial genomes in ME/CFS cases indicates that individuals of a certain haplogroup or carrying specific SNPs are more likely to exhibit certain neurological, inflammatory, and/or gastrointestinal symptoms. No increase in susceptibility to ME/CFS of individuals carrying particular mitochondrial genomes or SNPs was observed.

**Electronic supplementary material:**

The online version of this article (doi:10.1186/s12967-016-0771-6) contains supplementary material, which is available to authorized users.

## Background

The underlying cause of the disease known as Myalgic Encephalomyelitis (ME) or Chronic Fatigue Syndrome (CFS) is unknown, although a number of abnormalities have been detected in individuals with the illness. A substantial proportion of patients report becoming chronically ill after a flu-like illness, but others report a gradual onset. One favored hypothesis is that different types of insult result in the same outcome. As well as profound fatigue and post-exertional malaise, other symptoms that are common to many ME/CFS patients are neurological (difficulty concentrating, light sensitivity, sleep disturbances), inflammatory (such as flu-like symptoms, swollen lymph nodes, muscle pain) and gastrointestinal, sometimes including irritable bowel syndrome [1–4].

Mitochondrial dysfunction has been implicated in ME/CFS in multiple studies. One group has published three studies concerning ME/CFS that report impaired mitochondrial activity in neutrophils through an indirect assay of oxidative phosphorylation utilizing ATP measurement [5]. Interpretation of neutrophil studies is limited by the fact that they have fewer mitochondria than muscle cells or other types of blood cells. In contrast to the neutrophil analyses, two groups reported no detectable differences between the mitochondrial complex activities they assayed in peripheral blood mononuclear cells (PBMCs) of ME/CFS patients vs. controls [6, 7]. No ultrastructural abnormalities in mitochondria from CFS patients’ muscle were observed when an electron microscopic study was performed [8]. However, another group reported normal mitochondrial activity but decreased mitochondrial content in skeletal muscle of CFS patients [7]. Furthermore, two studies observed lower coenzyme Q content in blood cells or plasma of CFS patients [9, 10].

The literature on cardiac and/or skeletal muscle bioenergetic function analyzed by magnetic resonance spectroscopy in ME/CFS is also discordant, with some reports of abnormalities in CFS patients, while other reports indicate normal function [11–15]. It might be that only a fraction of cases exhibits abnormalities, which can hence go undetected when sample size is small. Either mitochondrial defects or problems in blood flow in muscles could result in anomalous findings in spectroscopic studies.

Little information is available concerning the mitochondrial genomes of individuals with ME/CFS. Particular variations in human mitochondrial genomes have been associated with different phenotypes such as climatic adaptation, high altitude tolerance and obesity, or increases or decreases in susceptibility or severity of medical problems such as diabetes, sepsis, stroke, Alzheimer’s and Parkinson’s disease, and hypertension, among others [16].

Human populations have inherited sets of polymorphisms representing different mitochondrial DNA (mtDNA) haplogroups. The effect of a nucleotide polymorphism may vary depending on the particular mitochondrial genome in which it arises. Haplogroup-level association testing is therefore valuable when analyzing the role of mtDNA polymorphisms in metabolic diseases. For example, such an approach was used to discover that mitochondrial haplogroup N9 confers resistance against type II diabetes in Asians [17]. Using individuals with European haplotypes, a recent study assessed whether or not two single nucleotide polymorphisms (SNPs) in a patient cohort were associated with symptoms and reported an association of one SNP with pain, chronic fatigue, and abnormal gastrointestinal motility [18]. Another study of mitochondrial genomes of 162 CFS cases carrying haplogroup H reported an association of disease status with a single SNP [19].

No reports have examined the degree of heteroplasmy (presence of more than one type of mitochondrial genome) in ME/CFS patients vs. controls. Somatic mutations in mitochondrial genomes can occur and are known to accumulate or to increase in effect during aging [16]. By obtaining the entire mitochondrial genome sequences from cells of ME/CFS patients and an age-matched cohort, it is possible to determine whether cases have accumulated more somatic mutations in mitochondrial DNA than expected. A recent examination of heteroplasmy in mitochondrial DNA from 1085 individuals found that 90 % carried at least one variant genome, and 20 % had sequence variants known to be pathogenic [20]. The frequency of pathogenic genomes in an individual can be examined through sequencing mitochondrial DNA at high depth, thus determining whether a threshold has been reached where disease has become manifest.

Heteroplasmic pathogenic mutations can also be transmitted through the germline, leading to mitochondrial disease if the proportion of mutant genomes becomes sufficiently high to impair function. For example, the m.3243A>G mutation can cause mitochondrial myopathy encephalopathy, lactic acidosis, and stroke (MELAS) when present at a frequency greater than 59 % [21].

We undertook a study of the mitochondrial genomes in a cohort of gender-and age-matched ME/CFS patients and controls of predominantly European origin. We performed next-generation sequencing to characterize the mtDNAs in the subjects with regard to haplotype, SNPs, and heteroplasmy. We carried out association testing to find whether any SNPs were significantly associated with ME/CFS status or with particular symptoms and their severity in the patient cohort.

## Methods

### Experimental design

#### Study population

CFI subjects were recruited at five different sites within the United States; Miami, Salt Lake City, Boston, New York City, and Sierra, Nevada and informed consent was obtained as described in Klimas et al. [22]. Cases were included in the study based on confirmed diagnosis by an expert clinician and based on either or both of the recognized ME/CFS case definitions, namely, the “1994 Fukuda criteria” and the “Canadian criteria”. The cohort was deliberately oversampled for patients with acute onset of the illness in the past 3 years. Gender, ethnicity, age (within 5 years)-matched healthy controls were specifically recruited for this study and were only included if they were both physically and mentally healthy. Time of sampling was also matched within 12 weeks from the sampling of cases and controls. Within a week of the questionnaire completion, blood was drawn, at which time the subjects underwent a full physical exam as well as filling out additional questionnaires to assess the severity of the illness on the day of sampling. All samples were processed similarly. Of the 405 subjects who participated in the CFI project, we received DNA from 193 patients with ME/CFS and 196 age and gender-matched healthy individuals from the CFI Biobank at the Duke Human Vaccine Institute (DHVI). DNA was prepared at DHVI from whole blood collected in Paxgene tubes (Qiagen, Germantown, MD).

#### Health questionnaires

In addition to demographic information, the Medical Outcomes Survey Short Form-36 (SF-36) and DePaul Symptom Questionnaire (DSQ) were administered to both cases and controls by the CFI physicians [22]. The SF-36 is a self-report general health survey of 36 items designed to compare the burden of disease on eight different aspects of well-being [23]. The DSQ is a self-report survey of the frequency and severity of 54 symptoms specifically associated with CFS, and covers various case definitions including the Fukuda et al. CFS criteria [24], Canadian Clinical criteria [25], and ME International Consensus criteria [26]. Only cases were surveyed by the DSQ. In the DSQ, frequency and severity of symptoms are scored from least to most on a scale from 0–4. An overall “distress” score is also calculated for each symptom on a scale from 0–16, by multiplying the frequency by severity scores [2, 27, 28]. In the CFI study, distress scores for the 54 symptoms were grouped into 10 functional clusters, and average cluster scores were calculated for each case [22].

#### DNA preparation and sequencing

Upon receipt from the Duke biobank, aliquots of the DNA samples were placed in 96-well plates before further processing. To avoid amplification of short nuclear mitochondrial copies, high-specificity long range PCR was performed, using NEB LongAmp^®^ Hot Start Taq DNA polymerase (New England Biolabs^®^, http://www.neb.com). The 50 uL PCR reaction consisted of the following mixture: 10 uL 5X LongAmp Taq Reaction Buffer, 1.5 uL of dNTPs (1 mM), 4 uL of each primer pairs (10 uM each, see below for details), 2 uL of template DNA, 2 uL of LongAmp Hot Start Taq DNA polymerase (2500 U/mL) and 30.5 uL of nuclease-free water. Amplification was performed with a thermocycler PTC-100 (MJ Research Inc., Waltham, MA, USA), starting with 30 s at 94 °C, followed by 30 cycles consisting of denaturation (20 s at 94 °C), annealing (1 min at 55 °C) and extension (10 min at 65 °C) and a final extension at 65 °C for 10 min.

The 16,569 bp mitochondrial genome was amplified using two primer pairs (F402: ATCTTTTGGCGGTATGCACTTT; R11428: GGCTTCGACATGGGCTTT and F8940: CCCCATACTAGTTATTATCGAAACC; R2818: GCCCCAACCGAAATTTTTAAT, from Lyons et al. [29] leading to two PCR products of 11,026 bp and 10,447 bp respectively, overlapping by almost 2500 bp. The overlap was necessary to prevent loss of end sequence information due to the Mu technology used in the subsequent library preparation.

The success of the PCR amplification was confirmed by electrophoresis and 5 uL of post-PCR reaction product was treated to degrade unused primers and nucleotides using USB^®^ HT ExoSAP-IT^®^ High-Throughput PCR Product Cleanup (Affymetrix, http://www.affymetrix.com) according to the manufacturer’s instructions.

The treated PCR products were diluted with 50 uL of nuclease-free water before Quant-IT™ PicoGreen^®^ dsDNA Assay Kit quantification (http://www.lifetechnologies.com) on a plate reader. The final mix of overlapping fragments was performed to obtain a final concentration of 0.2 ng/uL of each overlapping PCR product per sample before Illumina library preparation (http://www.illumina.com).

Library preparation was implemented by the Cornell Biotechnology Resource Center (BRC, http://www.biotech.cornell.edu/biotechnology-resource-center-brc) following the manufacturer’s instructions for the Nextera^®^ XT DNA Sample Preparation Kit (96 samples, FC-131-1096, http://www.illumina.com) and the Nextera XT Index Kits V2 Set A, B, C and D (FC-131-2001, FC-131-2002, FC-131-2003 and FC-131-2004, respectively, http://www.illumina.com) to be able to multiplex 384 samples during the sequencing reaction. After quality control for library size and amplification using Cornell BRC’s Fragment analyser, the Illumina MiSeq instrument generated paired-end reads (2 × 300 bp) with the MiSeq v3 kit. Three consecutive and identical runs were executed on the same library to increase coverage depth. Average mitochondrial DNA sequencing depth was 1497.3 ± 488.8 across patients and controls (Additional file 1: Figure S1).

### Bioinformatics and statistical analysis

#### Read mapping

Sequencing reads were mapped to the entire human reference genome (GRCh37) using the BWA-MEM alignment algorithm [30]. A number of methods were applied to filter out variation due to sequencing artifacts and errors. After mapping, a custom bash function was used to remove reads with more than three mismatches compared to the mitochondrial DNA reference sequence; the revised Cambridge Reference Sequence (rCRS) [31]. Duplicate reads were identified and removed using the Mark Duplicates function in the Picard tools suite. To avoid including reads sequenced from regions of the nuclear genome sharing high similarity with mitochondrial DNA (NUMTs), reads that did not map uniquely to the mitochondrial genome were discarded using SAMtools [32, 33]. SAMtools was also used to remove reads not mapping in a proper mate-pair to the mitochondrial genome using the command “samtools view –f 3 [bam file] chrM:1-16,569”. Strand-specific sequence alignment files were used to generate corresponding forward and reverse-strand pileup files with the SAMtools “mpileup” function. While generating pileup files, reads and bases with a PHRED quality scores less than 30 (0.001 chance of sequencing error) were discarded from the dataset using the “–q 30” and “–Q 30” flags. Strand-specific pileup files were then used to identify mtDNA consensus sequences and heteroplasmies as described below.

#### Consensus sequence identification

In order to measure associations with mtDNA SNPs and haplogroups, mtDNA consensus sequences were identified for each individual. From the pileup files, the major allele at each mtDNA base-pair position was called as the allele in the consensus sequence. Positions which did not have at least 10× sequencing coverage on each strand were not included.

#### Haplogroup classification

Individuals were classified into mtDNA haplogroups using the online Haplogrep tool [34] which infers mtDNA haplogroups based on the Phylotree database of observed human mtDNA variation [35]. A custom python script was used to convert the consensus sequences into the HSD input file format used by Haplogrep. All positions where the consensus allele for an individual did not match the revised rCRS were included in the HSD file [31]. In cases where the set of mtDNA mutations for an individual matched multiple haplogroups, the individual was assigned to the haplogroup with the highest overall Haplogrep ranking.

#### Haplogroup associations

Individual clinical phenotypes were measured using regression models in PLINK [36]. Only the five haplogroups that reached a minimum 5 % frequency threshold within our study population were tested for associations. In the PLINK regression analysis, each haplogroup was represented as a separate binary covariate where a value of one indicated that the given individual belonged to that haplogroup and a value of zero indicated they did not. For phenotypes that had more than two values, a linear regression was applied, while a logistic regression was applied to those with only two. A dummy SNP was included in order to perform the PLINK analysis, but had no effect on the significance values of each haplogroup-phenotype association. Multiple-test correction was applied to results using the Benjamini-Hochberg approach to calculate *q* values and significance of associations was determined using a 5 % FDR [37]. *P* values were sorted in ascending order and each divided by its percentile rank to obtain an estimated FDR.

#### Single-Marker analysis

Single-marker association testing was used to measure the association between SNPs and ME/CFS case/control status, as well as all symptoms surveyed in the SF-36 and DSQ as well as symptom clusters. Association testing was performed using the PLINK whole genome analysis [36]. Allele information at each position was obtained from the consensus sequence described previously. Before testing, mtDNA SNPs were filtered based on missingness, if data were missing from more than 10 % of individuals (the “—geno 0.1”), and allele frequency, excluding SNPs with frequency less than 5 % were (“—maf 0.05”). The sex, collection site, and age of each individual were included as covariates in the analyses. Sex was included using the “—sex” flag. In a separate covariate file, each collection site was assigned a binary variable indicating whether an individual’s information had been collected at that site. Age was included as a single variable in the covariate file. A logistic regression model (–logistic) was used to measure associations between mtDNA SNPs and the binary case–control status, while a linear model (–linear) was used to measure associations with quantitative SF-36 and DSQ symptoms. False Discovery Rate (FDR) was used, following the method of Benjamini and Hochberg [37] to correct for multiple testing by using the “—adjust” flag.

#### Heteroplasmy calling

Mitochondrial heteroplasmies can be present at very low frequencies where they are difficult to distinguish from sequencing error. A multi-step approach, based on previous studies with experimental and simulated data, was used to improve the chance of identifying true heteroplasmies. After read-mapping and quality control steps were applied to the data, as described in the “Read Mapping” section, data in the strand-specific pileup files was used to detect heteroplasmies. Minor alleles had to be present at a frequency of 1 % on both sequencing strands, and only mtDNA positions with at least 500× coverage on each strand were included. After using SAMtools to generate strand-specific pileup files, a custom Python script was used to collect and parse coverage and allele data. Double-strand validation was also used to check that the heteroplasmic alleles were present on both DNA strands at a frequency of at least 1 % [20, 38].

## Results

All subjects came from a large study supported by the Chronic Fatigue Initiative (CFI) [22, 39]. The subjects were diagnosed by physicians expert in ME/CFS and met either the Fukuda criteria [24] and/or the canadian consensus criteria [25] for diagnosis. Characteristics of subjects are shown in Table 1.

**Table 1:** Clinical characteristics of subjects surveyed

Age	Healthy controls		Patients			
	Number	Proportion females	Number	Proportion females	Acute onset	Duration of illness (>3 years)
21–30	25	0.6	23	0.57	14 (61 %)	11 (48 %)
31–40	22	0.48	20	0.65	14 (70 %)	15 (75 %)
41–50	34	0.71	40	0.75	32 (80 %)	27 (68 %)
51–60	71	0.76	59	0.63	51 (86 %)	45 (76 %)
61–75	44	0.7	51	0.8	43 (84 %)	47 (92 %)

### Causal mitochondrial disease variants

On average, there were 23.2 ± 11.3 SNPs in cases and 25.8 ± 12.4 in controls relative to the Cambridge reference sequence [31]. Polymorphisms observed in the consensus sequences of all individuals were cross-referenced with the MITOMAP database (http://www.mitomap.org/MITOMAP) of reported disease-associated mutations to determine whether any individuals harbored disease-causing mtDNA SNPs. Only one known mitochondrial-disease causing substitution was observed among the cases and controls. A T14484C substitution was found in a healthy control and is observed in individuals who develop Leber’s hereditary optic neuropathy (LHON) [40]. No other known disease-causing mtDNA variants were observed.

### Rare variant association analysis

In order to assess the effect of whole-mtDNA variation including common and rare variants, we compared the cumulative pathogenicity between controls and ME/CFS individuals. For each mtDNA variant in an individual, a raw combined annotation dependent depletion (CADD) pathogenicity score was obtained from the freely available CADD database (http://cadd.gs.washington.edu) [41]. An average CADD score was calculated for each individual by dividing the individual’s cumulative CADD score by the number of their variant sites. The distribution of average CADD scores was compared between cases and controls using the Kolmogorov–Smirnov test as implemented in R. There was no significant difference in the distribution of average CADD scores between cases and controls.

### Analysis of haplogroup with disease status

The vast majority of the individuals in the study (90.1 %) carried mtDNA haplogroups common to lineages of European descent, as expected because the cohort was >96 % white [22]. The most heavily represented haplogroups were H (39.1 %), U (15.4 %), T (10.6 %), K (9.1 %) and J (5.6 %). The remainder of the haplogroups represent lineages that spread through Asia and to the Americas (5.5 %) and those that are common to Africa (4.3 %).

The Fisher’s Exact Test was applied to compare the distributions of the haplogroups and showed no significant difference between cases and controls (Table 2). Additionally, we administered separate tests to each individual major haplogroup category. This revealed a nominally significant enrichment of haplogroup T (HgT) in controls versus cases that could suggest a potentially protective effect of HgT against CFS. However, the q-value (Benjamini and Hochberg corrected *p* value = 0.60) was not significant at a 5 % false discovery rate (FDR).

**Table 2:** Analysis of associations between CFS risk and mtDNA major haplogroups

Haplogroup	Case	Control	*p* value	*q* value*	Odds ratio	95 % CI
A	2	4	0.69	0.98	0.50	0.05 < OR < 3.56
B	2	2	1.00	1	1.02	0.07 < OR < 14.14
C	0	3	0.25	0.98	–	–
D	0	1	1.00	1	–	–
F	0	1	1.00	1	–	–
G	0	1	1.00	1	–	–
H	81	70	0.21	0.98	1.30	0.85 < OR < 2.00
I	4	7	0.54	0.98	0.57	0.12 < OR < 2.29
J	12	10	0.67	0.98	1.23	0.47 < OR < 3.27
K	20	15	0.38	0.98	1.39	0.65 < OR < 3.03
L	6	11	0.32	0.98	0.54	0.16 < OR < 1.63
M	2	1	0.62	0.98	2.04	0.11 < OR < 120.99
N	2	1	0.62	0.98	2.04	0.11 < OR < 120.99
R	1	0	0.50	0.98	–	–
T	14	28	0.03	0.6	0.47	0.22 < OR < 0.96
U	28	31	0.78	1	0.90	0.50 < OR < 1.63
V	6	3	0.33	0.98	2.06	0.43 < OR < 12.92
W	2	2	1.00	1	1.02	0.07 < OR < 14.14
X	4	2	0.45	0.98	2.05	0.29 < OR < 22.90
HV	7	3	0.22	0.98	2.42	0.54 < OR < 14.69

*q values were calculated using the Benjamini–Hochberg method for correcting for multiple testing of haplogroups

We also analyzed haplogroup distributions separately in males and females to account for sex-specific effects. We observed a nominally-significant enrichment of HgT among controls in females as above (Additional file 2: Table S1), but did not see any associations in males (Additional file 3: Table S2).

### Analysis of haplogroup with symptoms

After observing no major association with disease, haplogroup association testing was then applied to 270 different symptom scores collected from the SF-36 and DSQ surveys. Because many of the symptoms are related or dependent on each other, we did not apply multiple-test correction over the combined body of association tests. Only European haplogroups were present at a high enough frequency to be included in this analysis and HgJ, HgU, and HgH all had significant associations using a 5 % FDR. Performing individual association-testing and Benjamani-Hochberg *p* value correction for the sampling of 70 SNPs, we found six significant associations between mitochondrial haplogroups and clinical phenotypes assessed by the DePaul Questionnaire (Table 3) [23]. All of the significant associations were with symptoms related to joint pain, bloating, or “feeling dead” after exercise. Haplogroup J showed a protective effect against all metrics of joint pain and individuals belonging to haplogroup U reported less severe bloating and had lower bloating distress scores compared to other haplogroups. On the other hand, ME/CFS individuals with haplogroup H tended to be more susceptible to “feeling dead” after exercise than other haplogroups.

**Table 3:** Analysis of associations between ME/CFS symptoms and mtDNA haplogroups

Haplogroup	Phenotype	Haplogroup average	Non haplogroup average	*p* value	*q* value*
HgJ	Joint pain, frequency	1.08	2.17	0.00835	0.04177
HgJ	Joint pain, severity	1.00	1.96	0.00460	0.02300
HgJ	Joint pain, distress	2.33	5.83	0.00828	0.04144
HgU	Bloating, severity	0.82	1.43	0.00996	0.04982
HgU	Bloating, distress	1.50	3.66	0.00925	0.04626
HgH	"Feeling dead" after exercise, frequency	3.01	2.66	0.00779	0.03895

Haplogroup average describes the average phenotype value of all individuals within the given haplogroup

*q values were calculated using the Benjamini–Hochberg method for correcting for multiple testing of haplogroups. A higher phenotype value indicates higher frequency/severity. All significant phenotypes listed here were assessed using the DSQ and only include ME/CFS individuals

### Single-marker association analysis with disease

The majority of variable mtDNA sites (SNPs) in our dataset were singletons with only one individual carrying the variant alleles (Additional file 4: Figure S2), which were excluded from the association analysis. Seventy mtDNA SNPs met our minimum minor allele frequency threshold of 5 %, and were included in association tests with demographic and symptomatic variables associated with ME/CFS.

Single marker association testing was applied to measure SNP associations with the condition of individuals as either healthy controls or ME/CFS patients. Of the 70 SNPs, 10 reached nominal significance using an alpha threshold of 0.05, but none were significant with multiple test correction at a 5 % FDR (Additional file 5: Table S3). Within ME/CFS cases, we also measured SNP associations with acute or gradual onset of the disease and found no significant associations (Additional file 6: Table S4). In an effort to account for mtDNA genetic background and more accurately measure individual SNP effects, SNP associations were measured within the sub-group of individuals belonging to haplogroup H (HgH). Ideally, SNP associations would be measured within all haplogroups; however, given our sample size, HgH was the only haplogroup where analysis of association was feasible. Of the 151 HgH individuals in the study population for whom clinical data was available, none of the 18 SNPs meeting the 5 % frequency threshold had significant association with ME/CFS vs. healthy status.

The prevalence of CFS and fatigue-related syndromes has been observed to be higher in females than males. Additionally, because of the maternal inheritance pattern of mtDNA, mitochondrial variants are under sex-specific selective pressure that can lead to different fitness effects in males and females. To address possible sex differences, independent sex-stratified SNP association testing was performed, but revealed no associations (Additional file 7: Table S5 and Additional file 8: Table S6).

### Single-marker association analysis with symptoms

Single-marker association testing was then applied to symptom scores as done above for the haplogroup association analysis. We found eight mtDNA SNPs to be associated with 16 symptom categories at a 5 % FDR (Table 4). The significant SNPs were at mtDNA positions 150, 930, 1719, 3010, 5147, 16093, 16223, and 16519. SNPs 930 and 5147 shared a single association while 3010, 16093, and 16223 were each associated with two different symptoms. Five symptoms were associated with 16519, 150 was associated with four, and 1719 was associated with seven. Examination of quantile–quantile plots (QQ-plots) generated for each of the 21 significantly associated symptoms indicated that most did not exhibit any inflation of *p* values (Additional file 9: Figure S3).

**Table 4:** Analysis of associations between ME/CFS symptoms and mtDNA SNPs

Nucleotide position	Symptomatic allele	*p* value	*q* value*	Symptom	Type	Survey
150	T	0.000196	0.01373	Accomplished less emotional	–	SF-36
150	T	0.000308	0.02156	Emotional limitations	–	SF-36
150	T	8.94E-05	0.005944	Less time for work	–	SF-36
150	T	0.00048	0.03358	Didn’t work as carefully	–	SF-36
930	G	9.71E-05	0.006795	Difficulty performing work	–	SF-36
1719	A	3.80E-06	0.0002661	Inflammatory distress	Cluster	DePaul
1719	A	4.17E-05	0.002919	Flu-like symptoms	Distress	DePaul
1719	A	6.09E-05	0.004262	Chemical sensitivity	Distress	DePaul
1719	A	6.54E-05	0.00458	Neuro inflammatory distress	Cluster	DePaul
1719	A	0.000161	0.01129	Sensitivity to bright lights	Distress	DePaul
1719	A	0.000295	0.02068	Chemical sensitivity	Severity	DePaul
1719	A	0.000301	0.02108	Sensitivity to bright lights	Frequency	DePaul
3010	A	0.000173	0.01208	Sleep in day, awake all night	Frequency	DePaul
3010	A	0.000226	0.01582	Sleep in day, awake all night	Distress	DePaul
5147	G	0.001175	0.04114	Difficulty performing work	–	SF-36
16093	T	0.000206	0.0144	Accomplished less physical	–	SF-36
16093	T	0.000289	0.02022	Physical limitations	–	SF-36
16223	T	0.00076	0.0266	Sensitivity to bright lights	Frequency	DePaul
16223	T	0.000885	0.03098	Neuro inflammatory distress	Cluster	DePaul
16519	C	0.000125	0.008729	Gastrointestinal distress	Cluster	DePaul
16519	C	0.000135	0.009423	Bloating	Severity	DePaul
16519	C	0.000149	0.01043	Abdomen/stomach pain	Severity	DePaul
16519	C	0.000182	0.01274	Bloating	Frequency	DePaul
16519	C	0.000355	0.02482	Bloating	Distress	DePaul

*q values were calculated by the Benjamini–Hochberg correction applied to the number of SNPs included in the association test for each symptom

Chemical sensitivity refers to “Some smells, foods, medications, or chemicals make you feel sick”. The neuro inflammatory distress cluster combines “sensitivity to bright lights” and ”sensitivity to noise.” The inflammatory distress cluster combines these symptoms: sore throat, tender/sore lymph nodes, fever, flu-like symptoms, and chemical sensitivity. The gastrointestinal distress cluster combines bloating, abdomen/stomach pain, irritable bowel, and nausea. Subjects indicated whether they accomplished less due to emotional factors or because of physical limitations

**Fig. 1:**
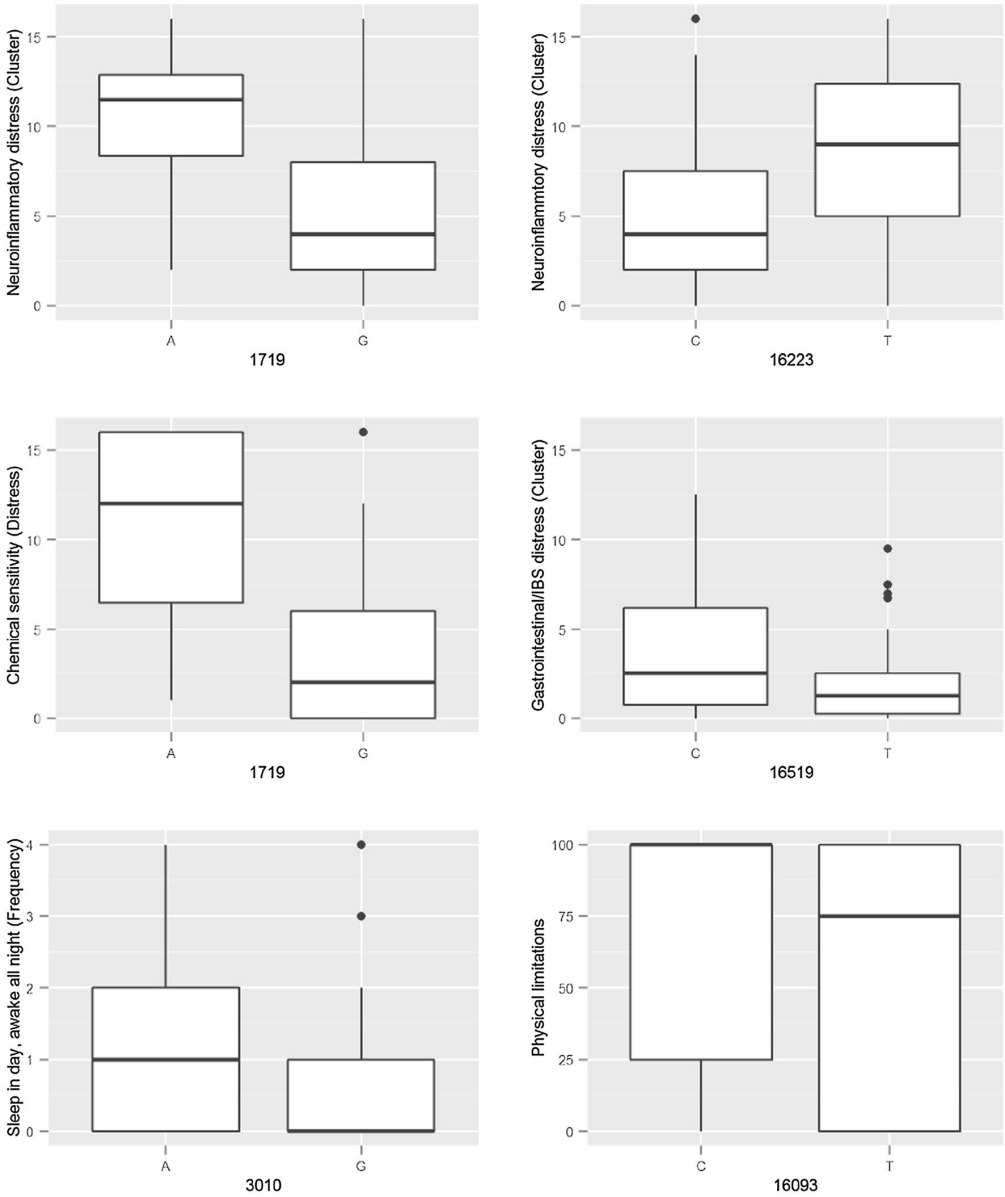
*Box plots* of SF-36 or DSQ symptom scores associated with mtDNA alleles. The *x-axis* shows mtDNA position and alleles while the *y-axis* shows symptom scores and is labelled with descriptions. *Scatter plots* of the six other significant associations can be found in Additional file 11: Figure S5. Some symptom descriptions were shortened for this figure (*less accomplishment*: accomplished less than you would like; *chemical sensitivity*: some smells, foods, medications, or chemicals induce sickness; *light sensitivity*: sensitivity to bright lights; *abdominal pain*: abdomen/stomach pain; *sleep disorder*: sleep all day and stay awake all night)

Allele 150T was associated with lower health scores in regards to emotional limitations, work ability, and accomplishments when surveyed across the ME/CFS patients using the SF-36 questionnaire. Both alleles 930G and 5147G were associated with greater difficulty performing work. The 1719A allele was associated with higher symptom scores in seven categories, all related to inflammation and neuroinflammation, as surveyed in ME/CFS patients using the DSQ. The two significant associations of 16223T with neuroinflammatory distress and frequency of sensitivity to bright lights overlapped with the same symptoms being associated with 1719A. Patients with 3010A suffered from higher frequency of and distress symptoms related to sleep disorder. Physical activities were more limited in patients with 16093T. The presence of 16519C was associated with more severe symptoms relating to gastrointestinal issues such as bloating and abdominal pain. Of the 24 total associations, there were four associations with cumulative distress clusters that had been calculated by grouping scores of functionally related DSQ symptoms [22] (Fig. 1 and Additional file 11: Figure S5).

### Heteroplasmy analysis

A mitochondrial heteroplasmy occurs when an individual carries copies of mtDNA with different alleles. Heteroplasmies can occur through somatic mutation or maternal inheritance and can be present at very low frequencies within the mtDNA population or at high frequencies. Mitochondria are thought to undergo a germline bottleneck that reduces the frequency of heteroplasmies transmitted from mother to child. However, if heteroplasmies reach a critical frequency within a tissue or individual, they can potentially have pathogenic effects. The accumulation of heteroplasmies during aging may also contribute to the variable age of onset observed in various energetic diseases.

**Fig. 2:**
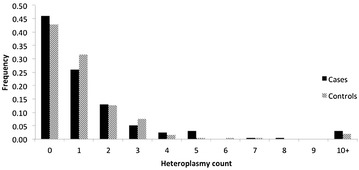
Heteroplasmy frequency spectrum within cohort. Heteroplasmy count (*x-axis*) refers to the number of heteroplasmies present in an individual. Frequency (*y-axis*) refers to the proportion of individuals with the given number of heteroplasmies in each case or control population

To investigate the role of heteroplasmy in CFS, we applied high coverage sequencing to identify and analyze heteroplasmies present in CFS patients. An average of 1.42 heteroplasmies per individual was observed in cases compared to only 1.28 in controls (Fig. 2). However, to account for the fact that the number of sites that met our criteria for calling heteroplasmies varied between samples, we calculated the number of heteroplasmies per usable site per individual (HPUI) and used that as metric for comparison. The Wilcoxon rank sum test with continuity correction was used to compare HPUI between cases and controls and showed no significant difference (*p* value = 0.93). The same test was used to compare the distribution of HPUI between patients who experienced acute onset of ME/CFS versus those who experienced onset, and no significant difference was observed (*p* value = 0.30, Additional file 10: Figure S4). Since cytokine profiles of ME/CFS patients have been observed to differ with the duration of the disease [39], HPUI was also compared between individuals who had experienced ME/CFS for 3 years or less and those who had endured it for more than 3 years. Again, the Wilcoxon rank sum test showed no significant difference in HPUI between groups (*p* value = 0.84, Additional file 12: Figure S6).

**Fig. 3:**
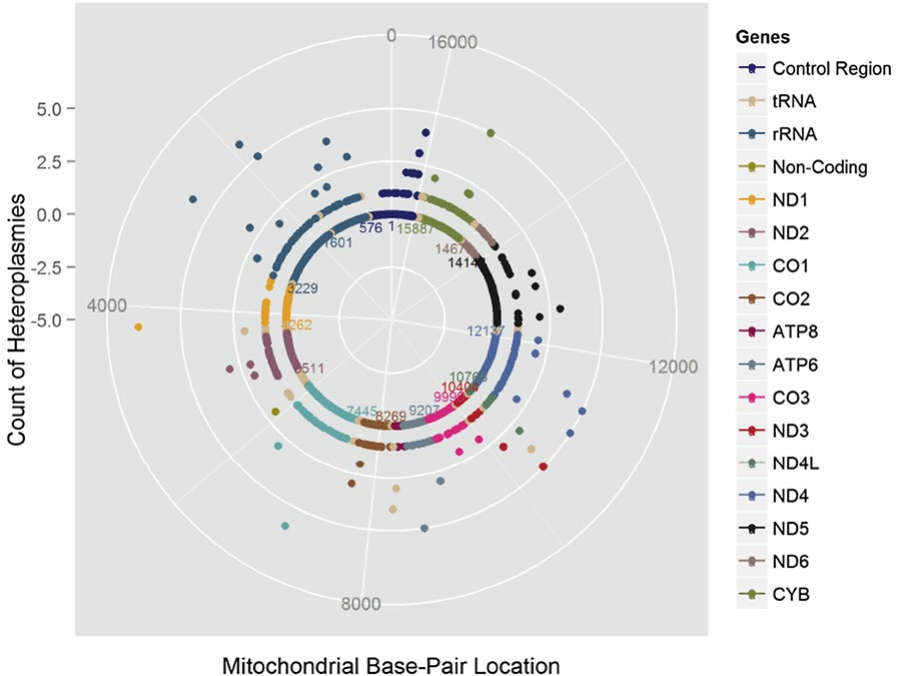
*Solarplot* representing the location and frequency of heteroplasmies in the CFI cohort. The *innermost ring* represents the mtDNA sequence and each *color* coding corresponds to a different functional region. Each *dot* outside of the *centermost ring* represents a position where there is a heteroplasmy. The distance of the *dot* from the *innermost ring* indicates how many people in the dataset carry that heteroplasmy

Comparison of the frequency of heteroplasmic alleles revealed very few heteroplasmies occurred in either cases or controls. Of the 406 different heteroplasmies observed between cases and controls, the average heteroplasmy was present in only 1.23 ± 0.85 individuals and 353 heteroplasmies were only found in single individuals (Fig. 3). The most common heteroplasmy was present in seven individuals.

## Discussion

Our sequencing of the entire mitochondrial genome of patients revealed that no subjects who were included in this cohort have genetic mitochondrial disease due to known mtDNA mutations that was mis-identified as ME/CFS. Therefore, the expert physicians who selected the cases for inclusion did not erroneously include any individuals with a known mitochondrial disease.

Our study examined associations between mtDNA variation and demographic variables such as ME/CFS status, acute onset, and illness duration. We found a correlation between haplogroup and some symptoms related to joint pain, bloating and dead/heavy feeling after exercise. Most studies of mitochondrial DNA and disease pertain to the increased or decreased susceptibility to the disease depending on haplogroup or variant [42], rather than differences in type of symptoms or their severity. However, there are some diseases in which a haplogroup has been associated not with the chance of acquiring a disease, but instead the outcome once someone has been diagnosed. For example, haplogroup affects survival of severe sepsis [43] and progression of AIDS [44].

Measuring mtDNA SNP associations with ME/CFS case–control status did not reveal any associations significant at a 5 % FDR, though 10 SNPs reached nominal significance. Examination of these 10 SNPs revealed nine of them to be marker polymorphisms for HgT, and the tenth to be a marker for five other recognized sub-haplogroups within T. The finding is likely due to the fact that haplogroup association analysis found that more controls than ME/CFS subjects carried HgT in our cohort. A larger cohort will be needed to determine whether this haplotype is somewhat protective against ME/CFS. With regard to HgT, a molecular analysis of cybrid cells with identical nuclear backgrounds and different mtDNA populations indicated that HgT cybrids had a higher survival rate and were more tolerant of oxidative stress when compared to the more common European haplogroup H [45].

One previous study examined mitochondrial DNA SNPs at 3010 and 16519 in 162 CFS patients vs. controls. Boles et al. [18] found an enrichment of 16519T in CFS cases. However, in our study, we did not observe a significant association of 16519T or any other polymorphism to CFS vs. control status. Instead, among individuals with ME/CFS, we observed a significant association of 16519C with gastrointestinal symptoms. Furthermore, the alleles we found to be associated with more severe ME/CFS symptoms were 16519C and 3010A.

The variation in symptom constellation that occurs in ME/CFS could be due to genetic variation in individuals, rather than differences in underlying cause of the disease. Measurement of associations between haplogroups or mtDNA SNPs and symptom scores of ME/CFS patients collected through SF-36 and DSQ surveys revealed 24 significant associations at a 5 % FDR. Although we undertook this study because some of the major symptoms of ME/CFS could theoretically be due to mitochondrial dysfunction, we detected no mitochondrial DNA SNP associations with the major symptom clusters fatigue/post-exertional malaise, cognitive difficulties, autonomic dysfunction, endocrine abnormalities, and pain. Instead, we observed associations with neurological, inflammatory, and gastrointestinal symptoms and/or their severity, as well as correlations with the patients’ abilities to do work. It will be important to utilize a completely independent cohort to determine whether the correlations we have detected can be replicated.

Our study provides hints that at least some of the apparent sub-types in ME/CFS could be due to differences between DNA sequences in the patients, even though we have no evidence that particular mitochondrial genomes lead to increased susceptibility to the disease itself. Although a sample size of 389 is quite large in comparison to most ME/CFS studies, further analysis of the mitochondrial and nuclear genomes in a much larger population of patients will be needed to discover how genetic variation affects illness severity, progression, and symptom clusters.

## Conclusions

We did not observe a significant association of mitochondrial DNA genome variation with either susceptibility or resistance to ME/CFS. We did not detect any significant difference in level of heteroplasmy between cases and controls. Using a cohort of 193 ME/CFS cases and 196 controls, at 5 % FDR we observed eight mtDNA SNPs to be associated with 16 symptom categories and three haplogroups associated with six symptom categories, suggesting that the mitochondrial genome of an individual with ME/CFS can affect the type and severity of particular symptoms.

## Additional files

10.1186/s12967-016-0771-6 Average mitochondrial DNA sequencing depth across all case and control individuals.
10.1186/s12967-016-0771-6 Association analysis of CFS risk and mtDNA haplogroup in females.
10.1186/s12967-016-0771-6 Association analysis of CFS risk with mtDNA haplogroups in males. ure**. S2.** 10.1186/s12967-016-0771-6 Site frequency spectrum of variant mtDNA positions in the CFS cohort.
10.1186/s12967-016-0771-6 Association analysis of mtDNA SNPs within ME/CFS status.
10.1186/s12967-016-0771-6 Association analysis of mtDNA SNPs with acute vs gradual onset in ME/CFS patients.
10.1186/s12967-016-0771-6 Association analysis of mtDNA SNPs in males.
10.1186/s12967-016-0771-6 Association analysis of mtDNA SNPs in females.
10.1186/s12967-016-0771-6 Quantile–quantile plots of symptoms significantly associated with mtDNA SNPs.
10.1186/s12967-016-0771-6 Boxplots of SF-36/DSQ symptom scores associated with mtDNA alleles.
10.1186/s12967-016-0771-6 Comparison of HPUI distribution between individuals with acute and gradual CFS onset.
10.1186/s12967-016-0771-6 Comparison of HPUI distribution between individuals who have experienced ME/CFS for less than 3 years and more than 3 years.

## References

[1] Hutchinson CV, Maltby J, Badham SP, Jason LA (2014). Vision-related symptoms as a clinical feature of chronic fatigue syndrome/myalgic encephalomyelitis? Evidence from the DePaul Symptom Questionnaire. Br J Ophthalmol.

[2] Jason LA, Evans M, Porter N, Brown M, Brown A, Hunnell J (2010). The development of a revised Canadian myalgic encephalomyelitis-chronic fatigue syndrome case definition. Am J Biochem Biotechnol.

[3] Aaron LA, Burke MM, Buchwald D (2000). Overlapping conditions among patients with chronic fatigue syndrome, fibromyalgia, and temporomandibular disorder. Arch Int Med.

[4] Komaroff AL, Buchwald D (1991). Symptoms and signs of chronic fatigue syndrome. Rev Infect Dis.

[5] Myhill S, Booth NE, McLaren-Howard J (2013). Targeting mitochondrial dysfunction in the treatment of myalgic encephalomyelitis/chronic fatigue syndrome (ME/CFS)—a clinical audit. Int J Clin Exp Med.

[6] Vermeulen RC, Kurk RM, Visser FC, Sluiter W, Scholte HR (2010). Patients with chronic fatigue syndrome performed worse than controls in a controlled repeated exercise study despite a normal oxidative phosphorylation capacity. J Transl Med.

[7] Smits B, van den Heuvel L, Knoop H, Kusters B, Janssen A, Borm G (2011). Mitochondrial enzymes discriminate between mitochondrial disorders and chronic fatigue syndrome. Mitochondrion.

[8] Plioplys AV, Plioplys S (1995). Electron-microscopic investigation of muscle mitochondria in chronic fatigue syndrome. Neuropsychobiology.

[9] Castro-Marrero J, Cordero MD, Saez-Francas N, Jimenez-Gutierrez C, Aguilar-Montilla FJ, Aliste L (2013). Could mitochondrial dysfunction be a differentiating marker between chronic fatigue syndrome and fibromyalgia?. Antiox. Redox Sig.

[10] Maes M, Mihaylova I, Kubera M, Uytterhoeven M, Vrydags N, Bosmans E (2009). Coenzyme Q10 deficiency in myalgic encephalomyelitis/chronic fatigue syndrome (ME/CFS) is related to fatigue, autonomic and neurocognitive symptoms and is another risk factor explaining the early mortality in ME/CFS due to cardiovascular disorder. Neuro endocrinol Lett.

[11] Hollingsworth KG, Jones DE, Taylor R, Blamire AM, Newton JL (2010). Impaired cardiovascular response to standing in chronic fatigue syndrome. Eur J Clin Invest.

[12] Lane RJ, Barrett MC, Taylor DJ, Kemp GJ, Lodi R (1998). Heterogeneity in chronic fatigue syndrome: evidence from magnetic resonance spectroscopy of muscle. Neuromuscul Dis.

[13] Wong R, Lopaschuk G, Zhu G, Walker D, Catellier D, Burton D (1992). Skeletal muscle metabolism in the chronic fatigue syndrome. In vivo assessment by 31P nuclear magnetic resonance spectroscopy. Chest.

[14] McCully KK, Smith S, Rajaei S, Leigh JS, Natelson BH (2003). Blood flow and muscle metabolism in chronic fatigue syndrome. Clin Sci.

[15] McCully KK, Smith S, Rajaei S, Leigh JS, Natelson BH (2004). Muscle metabolism with blood flow restriction in chronic fatigue syndrome. J Appl Phys.

[16] Wallace DC (2010). Mitochondrial DNA mutations in disease and aging. Environ Mol Mutagen.

[17] Fuku N, Park KS, Yamada Y, Nishigaki Y, Cho YM, Matsuo H (2007). Mitochondrial haplogroup N9a confers resistance against type 2 diabetes in Asians. Am J Hum Genet.

[18] Boles RG, Hornung HA, Moody AE, Ortiz TB, Wong SA, Eggington JM (2015). Hurt, tired and queasy: specific variants in the ATPase domain of the TRAP1 mitochondrial chaperone are associated with common, chronic “functional” symptomatology including pain, fatigue and gastrointestinal dysmotility. Mitochondrion.

[19] Boles RG, Zaki EA, Kerr JR, Das K, Biswas S, Gardner A (2015). Increased prevalence of two mitochondrial DNA polymorphisms in functional disease: are we describing different parts of an energy-depleted elephant?. Mitochondrion.

[20] Ye K, Lu J, Ma F, Keinan A, Gu Z (2014). Extensive pathogenicity of mitochondrial heteroplasmy in healthy human individuals. Proc Natl Acad Sci USA.

[21] de Laat P, Koene S, van den Heuvel LP, Rodenburg RJ, Janssen MC, Smeitink JA (2012). Clinical features and heteroplasmy in blood, urine and saliva in 34 Dutch families carrying the m.3243A>G mutation. J Inherit Metab Dis.

[22] Klimas NG, Ironson G, Carter A, Balbin E, Bateman L, Felsenstein D (2015). Findings from a clinical and laboratory database developed for discovery of pathogenic mechanisms in myalgic encephalomyelitis/chronic fatigue syndrome. Fatigue.

[23] Ware JE, Kosinski M, Bayliss MS, McHorney CA, Rogers WH, Raczek A (1995). Comparison of methods for the scoring and statistical analysis of SF-36 health profile and summary measures: summary of results from the medical outcomes study. Med Care.

[24] Fukuda K, Straus SE, Hickie I, Sharpe MC, Dobbins JG, Komaroff A (1994). The chronic fatigue syndrome: a comprehensive approach to its definition and study. International Chronic Fatigue Syndrome Study Group. Ann Intern Med.

[25] Carruthers BM, Jain AK, DeMeirleir KL, Peterson DL, Klimas NG, Lerner AM (2003). Myalgic encephalomyelitis/chronic fatigue syndrome: clinical working case definition, diagnostic and treatments protocols. J Chronic Fatigue Synd.

[26] Carruthers BM, van de Sande MI, De Meirleir KL, Klimas NG, Broderick G, Mitchell T (2011). Myalgic encephalomyelitis: international Consensus Criteria. J Int Med.

[27] Jason LA, So S, Brown AA, Sunnquist M, Evans ME (2015). Test–retest reliability of the DePaul symptom questionnaire. Fatigue.

[28] Brown AA, Jason LA (2014). Validating a measure of myalgic encephalomyelitis/chronic fatigue syndrome symptomatology. Fatigue.

[29] Lyons EA, Scheible MK, Sturk-Andreaggi K, Irwin JA, Just RS (2013). A high-throughput Sanger strategy for human mitochondrial genome sequencing. BMC Genomics.

[30] Li H, Durbin R (2011). Inference of human population history from individual whole-genome sequences. Nature.

[31] Bandelt HJ, Kloss-Brandstatter A, Richards MB, Yao YG, Logan I (2014). The case for the continuing use of the revised cambridge reference sequence (rCRS) and the standardization of notation in human mitochondrial DNA studies. J Hum Genet.

[32] Dayama G, Emery SB, Kidd JM, Mills RE (2014). The genomic landscape of polymorphic human nuclear mitochondrial insertions. Nuc Acids Res.

[33] Li H, Handsaker B, Wysoker A, Fennell T, Ruan J, Homer N (2009). The sequence alignment/map format and SAMtools. Bioinformatics.

[34] Kloss-Brandstatter A, Pacher D, Schonherr S, Weissensteiner H, Binna R, Specht G (2011). HaploGrep: a fast and reliable algorithm for automatic classification of mitochondrial DNA haplogroups. Hum Mutat.

[35] van Oven M, Kayser M (2009). Updated comprehensive phylogenetic tree of global human mitochondrial DNA variation. Hum Mutat.

[36] Purcell S, Neale B, Todd-Brown K, Thomas L, Ferreira MA, Bender D (2007). PLINK: a tool set for whole-genome association and population-based linkage analyses. Am J Human Genet.

[37] Benjamini Y, Hochberg Y (1995). Controlling the false discovery rate—a practical and powerful approach to multiple testing. J Roy Stat Soc B Met.

[38] Goto H, Dickins B, Afgan E, Paul IM, Taylor J, Makova KD (2011). Dynamics of mitochondrial heteroplasmy in three families investigated via a repeatable re-sequencing study. Genome Biol.

[39] Hornig M, Montoya JG, Klimas NG, Levine S, Felsenstein D, Bateman L (2015). Distinct plasma immune signatures in ME/CFS are present early in the course of illness. Sci Adv.

[40] Chinnery PF, Brown DT, Andrews RM, Singh-Kler R, Riordan-Eva P, Lindley J (2001). The mitochondrial ND6 gene is a hot spot for mutations that cause Leber’s hereditary optic neuropathy. Brain.

[41] Kircher M, Witten DM, Jain P, O’Roak BJ, Cooper GM, Shendure J (2014). A general framework for estimating the relative pathogenicity of human genetic variants. Nature Genet.

[42] Urzúa-Traslaviña CG, Moreno-Treviño MG, Martínez-Treviño DA, Barrera-Saldaña HA, León-Cachón RBR (2014). Relationship of mitochondrial DNA haplogroups with complex diseases. J Genet Genome Res.

[43] Baudouin SV, Saunders D, Tiangyou W, Elson JL, Poynter J, Pyle A (2005). Mitochondrial DNA and survival after sepsis: a prospective study. Lancet.

[44] Hart AB, Samuels DC, Hulgan T (2013). The other genome: a systematic review of studies of mitochondrial DNA haplogroups and outcomes of HIV infection and antiretroviral therapy. AIDS Rev.

[45] Mueller EE, Brunner SM, Mayr JA, Stanger O, Sperl W, Kofler B (2012). Functional differences between mitochondrial haplogroup T and haplogroup H in HEK293 cybrid cells. PLoS One.

