# Supplementary material for: Mitochondrial DNA variants correlate with symptoms in myalgic encephalomyelitis/chronic fatigue syndrome
Source: J Transl Med. 2016 Jan 20;14:19. doi: 10.1186/s12967-016-0771-6 (PMC4719218; doi:10.1186/s12967-016-0771-6)
Supplement: Supplementary file 1 — 10.1186/s12967-016-0771-6 Average mitochondrial DNA sequencing depth across all case and control individuals. [file 12967_2016_771_MOESM1_ESM.docx]

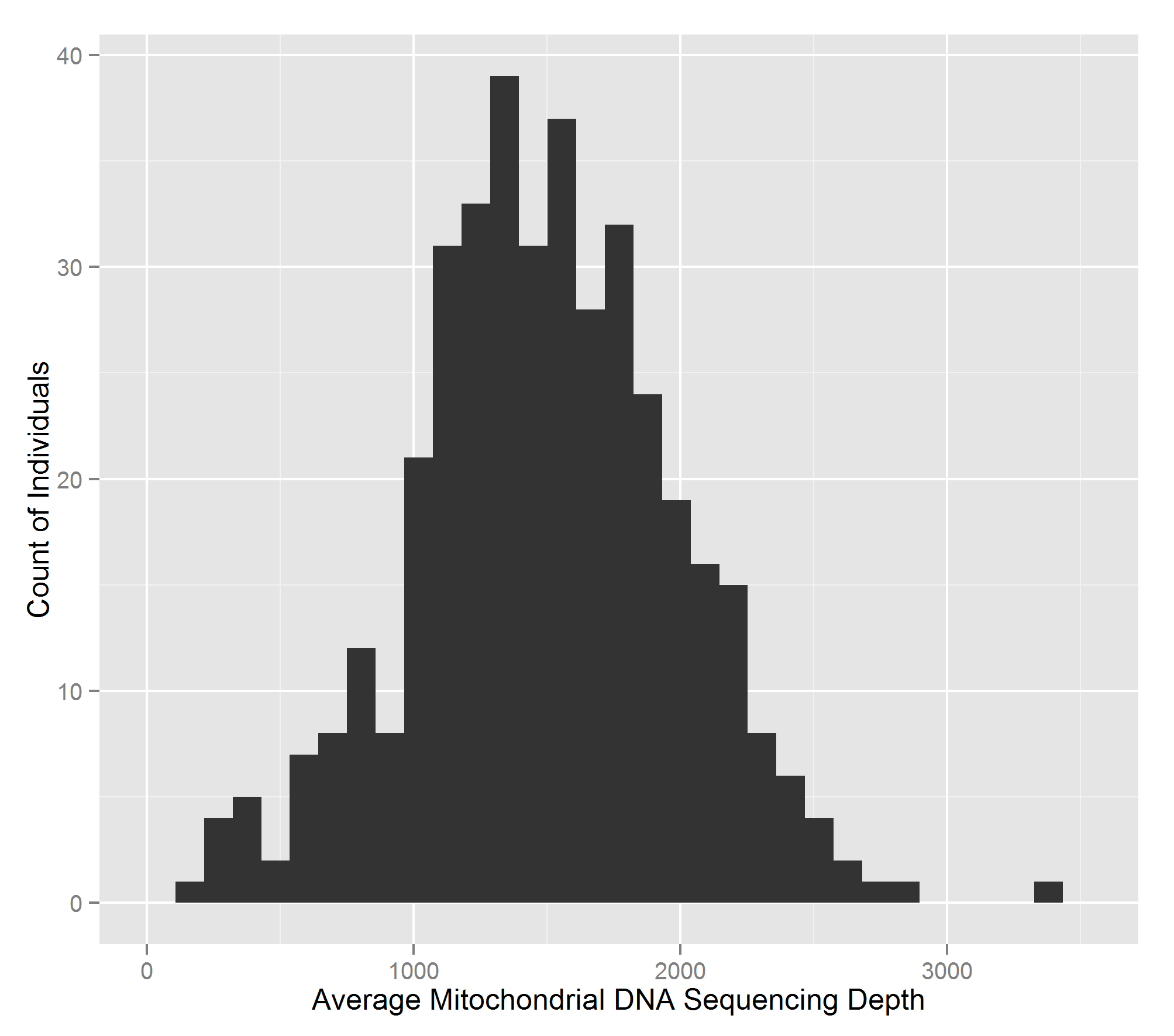


Additional file 1: Fig. S1. Average mitochondrial DNA sequencing depth across all case and control individuals.
