# Supplementary material for: Mitochondrial DNA variants correlate with symptoms in myalgic encephalomyelitis/chronic fatigue syndrome
Source: J Transl Med. 2016 Jan 20;14:19. doi: 10.1186/s12967-016-0771-6 (PMC4719218; doi:10.1186/s12967-016-0771-6)
Supplement: Supplementary file 2 — 10.1186/s12967-016-0771-6 Association analysis of CFS risk and mtDNA haplogroup in females. [file 12967_2016_771_MOESM2_ESM.docx]

**Additional file 2: Table S1. Association analysis of CFS risk and mtDNA haplogroup in females.**

|  | Case | Control | P value | Odds Ratio | 95% CI |
| --- | --- | --- | --- | --- | --- |
| A | 2 | 4 | 0.68 | 0.51 | 0.05<OR<3.62 |
| B | 2 | 2 | 1 | 1.03 | 0.07<OR<14.40 |
| C | 0 | 2 | 0.5 | 0 | 0.00<OR<5.48 |
| D | 0 | 1 | 1 | - | - |
| F | 0 | 1 | 1 | - | - |
| G | 0 | 1 | 1 | - | - |
| H | 53 | 46 | 0.31 | 1.31 | 0.77<OR<2.21 |
| I | 3 | 2 | 0.68 | 1.55 | 0.18<OR<18.89 |
| J | 10 | 9 | 0.82 | 1.16 | 0.41<OR<3.33 |
| K | 14 | 9 | 0.28 | 1.67 | 0.65<OR<4.54 |
| L | 4 | 9 | 0.26 | 0.44 | 0.10<OR<1.63 |
| M | 1 | 1 | 1 | 1.03 | 0.01<OR<81.39 |
| N | 2 | 1 | 0.62 | 2.07 | 0.11<OR<123.25 |
| R | 1 | 0 | 0.49 | - | - |
| T | 9 | 20 | 0.05 | 0.43 | 0.16<OR<1.03 |
| U | 18 | 23 | 0.5 | 0.78 | 0.37<OR<1.59 |
| V | 5 | 2 | 0.28 | 2.63 | 0.42<OR<28.05 |
| W | 2 | 2 | 1 | 1.03 | 0.07<OR<14.40 |
| X | 2 | 2 | 1 | 1.03 | 0.07<OR<14.40 |
| HV | 6 | 1 | 0.06 | 6.39 | 0.76<OR<297.08 |
