# Supplementary material for: Mitochondrial DNA variants correlate with symptoms in myalgic encephalomyelitis/chronic fatigue syndrome
Source: J Transl Med. 2016 Jan 20;14:19. doi: 10.1186/s12967-016-0771-6 (PMC4719218; doi:10.1186/s12967-016-0771-6)
Supplement: Supplementary file 3 — 10.1186/s12967-016-0771-6 Association analysis of CFS risk with mtDNA haplogroups in males. [file 12967_2016_771_MOESM3_ESM.docx]

Additional file 3: Table S2. Association analysis of CFS risk with mtDNA haplogroups in males.

|  | Case | Control | P value | Odds Ratio | 95% CI |
| --- | --- | --- | --- | --- | --- |
| C | 0 | 1 | 0.5 | - | 0.00<OR<38.34 |
| H | 28 | 24 | 0.58 | 1.28 | 0.58<OR<2.84 |
| I | 1 | 5 | 0.11 | 0.19 | 0.00<OR<1.73 |
| J | 2 | 1 | 1 | 1.99 | 0.10<OR<119.94 |
| K | 6 | 6 | 1 | 0.98 | 0.24<OR<3.93 |
| L | 2 | 2 | 1 | 0.98 | 0.07<OR<13.99 |
| M | 1 | 0 | 1 | - | - |
| T | 5 | 8 | 0.39 | 0.58 | 0.14<OR<2.17 |
| U | 10 | 8 | 0.8 | 1.27 | 0.41<OR<4.05 |
| V | 1 | 1 | 1 | 0.98 | 0.01<OR<78.43 |
| X | 2 | 0 | 0.5 | - | - |
| HV | 1 | 2 | 0.62 | 0.49 | 0.01<OR<9.57 |
