# Supplementary material for: Mitochondrial DNA variants correlate with symptoms in myalgic encephalomyelitis/chronic fatigue syndrome
Source: J Transl Med. 2016 Jan 20;14:19. doi: 10.1186/s12967-016-0771-6 (PMC4719218; doi:10.1186/s12967-016-0771-6)
Supplement: Supplementary file 4 — ure. S2. 10.1186/s12967-016-0771-6 Site frequency spectrum of variant mtDNA positions in the CFS cohort. [file 12967_2016_771_MOESM4_ESM.docx]

Additional file 4: Fig. S2. Site frequency spectrum of variant mtDNA positions in the CFS cohort. Minor allele count (x-axis) refers to the number of individuals possessing the minor allele at a given mtDNA position. Frequency (y-axis) indicates the proportion of mtDNA positions that had the given number of minor alleles.
