# Supplementary material for: Mitochondrial DNA variants correlate with symptoms in myalgic encephalomyelitis/chronic fatigue syndrome
Source: J Transl Med. 2016 Jan 20;14:19. doi: 10.1186/s12967-016-0771-6 (PMC4719218; doi:10.1186/s12967-016-0771-6)
Supplement: Supplementary file 5 — 10.1186/s12967-016-0771-6 Association analysis of mtDNA SNPs within ME/CFS status. [file 12967_2016_771_MOESM5_ESM.docx]

**Additional file 5: Table S3. Association analysis of mtDNA SNPs within ME/CFS status.**

| Base-pair | Minor allele | Nominal p-value | Odds Ratio | Benjamini-Hochberg FDR |
| --- | --- | --- | --- | --- |
| 709 | A | 0.01067 | 0.4751 | 0.3274 |
| 8697 | A | 0.01083 | 0.406 | 0.3274 |
| 10463 | C | 0.02113 | 0.4626 | 0.3274 |
| 11914 | A | 0.03402 | 0.417 | 0.3274 |
| 11719 | G | 0.03553 | 1.55 | 0.3274 |
| 1888 | A | 0.04332 | 0.5055 | 0.3274 |
| 4917 | G | 0.04592 | 0.5069 | 0.3274 |
| 13368 | A | 0.04778 | 0.5032 | 0.3274 |
| 15607 | G | 0.04778 | 0.5032 | 0.3274 |
| 15928 | A | 0.04778 | 0.5032 | 0.3274 |
| 16294 | T | 0.05145 | 0.5329 | 0.3274 |
| 15301 | A | 0.0637 | 0.3993 | 0.3518 |
| 195 | C | 0.07071 | 1.663 | 0.3518 |
| 14766 | C | 0.07223 | 1.456 | 0.3518 |
| 10873 | C | 0.07623 | 0.4562 | 0.3518 |
| 4216 | C | 0.09611 | 0.6361 | 0.3518 |
| 15043 | A | 0.1015 | 0.4632 | 0.3518 |
| 9540 | C | 0.1037 | 0.4839 | 0.3518 |
| 16189 | C | 0.1044 | 0.6369 | 0.3518 |
| 12705 | T | 0.1049 | 0.6087 | 0.3518 |
| 14905 | A | 0.1055 | 0.5825 | 0.3518 |
| 5147 | A | 0.1108 | 0.4862 | 0.3526 |
| 11251 | G | 0.1188 | 0.648 | 0.3601 |
| 8701 | G | 0.1243 | 0.4998 | 0.3601 |
| 14233 | G | 0.1337 | 0.5676 | 0.3601 |
| 11812 | G | 0.1338 | 0.5678 | 0.3601 |
| 146 | C | 0.1846 | 0.645 | 0.4686 |
| 15452 | A | 0.1874 | 0.6914 | 0.4686 |
| 2706 | A | 0.2105 | 1.299 | 0.508 |
| 3480 | G | 0.2316 | 1.563 | 0.5202 |
| 16126 | C | 0.2407 | 0.7222 | 0.5202 |
| 1189 | C | 0.2411 | 1.575 | 0.5202 |
| 16223 | T | 0.2476 | 0.7063 | 0.5202 |
| 7028 | C | 0.2566 | 1.274 | 0.5202 |
| 73 | A | 0.2601 | 1.263 | 0.5202 |
| 14798 | C | 0.2753 | 1.413 | 0.5353 |
| 16362 | C | 0.3068 | 1.444 | 0.5804 |
| 3010 | A | 0.3495 | 1.271 | 0.6371 |

**Additional file 5:** **Table S3 (Continued). Association analysis of mtDNA SNPs within ME/CFS status.**

| Base-pair | Minor allele | Nominal p-value | Odds Ratio | Benjamini-Hochberg FDR |
| --- | --- | --- | --- | --- |
| 11299 | C | 0.355 | 1.393 | 0.6371 |
| 10550 | G | 0.3647 | 1.383 | 0.6381 |
| 9055 | A | 0.387 | 1.352 | 0.6416 |
| 16224 | C | 0.3996 | 1.357 | 0.6416 |
| 16270 | T | 0.4022 | 0.7197 | 0.6416 |
| 14167 | T | 0.4033 | 1.354 | 0.6416 |
| 12612 | G | 0.4485 | 1.391 | 0.6977 |
| 16311 | C | 0.4678 | 1.232 | 0.6987 |
| 9698 | C | 0.4691 | 1.29 | 0.6987 |
| 15924 | G | 0.5119 | 0.7775 | 0.7465 |
| 497 | T | 0.555 | 1.292 | 0.7852 |
| 16093 | C | 0.5628 | 0.7691 | 0.7852 |
| 16069 | T | 0.5721 | 1.284 | 0.7852 |
| 16519 | T | 0.6181 | 1.118 | 0.8101 |
| 13708 | A | 0.6247 | 1.206 | 0.8101 |
| 3197 | C | 0.6283 | 0.8204 | 0.8101 |
| 13617 | C | 0.6396 | 0.8336 | 0.8101 |
| 16183 | C | 0.6705 | 0.8207 | 0.8101 |
| 10398 | G | 0.6728 | 0.9025 | 0.8101 |
| 930 | A | 0.6917 | 0.8453 | 0.8101 |
| 489 | C | 0.7039 | 0.8647 | 0.8101 |
| 16192 | T | 0.7168 | 0.8456 | 0.8101 |
| 9477 | A | 0.7258 | 0.8748 | 0.8101 |
| 12308 | G | 0.7291 | 1.087 | 0.8101 |
| 12372 | A | 0.7291 | 1.087 | 0.8101 |
| 11467 | G | 0.7517 | 1.079 | 0.8221 |
| 1719 | A | 0.8081 | 1.11 | 0.8672 |
| 150 | T | 0.8176 | 0.924 | 0.8672 |
| 1811 | G | 0.8472 | 1.058 | 0.8852 |
| 152 | C | 0.8755 | 0.9624 | 0.9012 |
| 16278 | T | 0.9247 | 1.038 | 0.9346 |
| 16304 | C | 0.9346 | 0.9705 | 0.9346 |
