# Supplementary material for: Mitochondrial DNA variants correlate with symptoms in myalgic encephalomyelitis/chronic fatigue syndrome
Source: J Transl Med. 2016 Jan 20;14:19. doi: 10.1186/s12967-016-0771-6 (PMC4719218; doi:10.1186/s12967-016-0771-6)
Supplement: Supplementary file 6 — 10.1186/s12967-016-0771-6 Association analysis of mtDNA SNPs with acute vs gradual onset in ME/CFS patients. [file 12967_2016_771_MOESM6_ESM.docx]

**Additional file 6: Table S4. Association analysis of mtDNA SNPs with acute vs gradual onset in ME/CFS patients.**

| Base-pair | Minor allele | Nominal p-value | Odds Ratio | Benjamini-Hochberg FDR |
| --- | --- | --- | --- | --- |
| 5147 | A | 0.06307 | 0.2201 | 0.9721 |
| 146 | C | 0.066 | 7.229 | 0.9721 |
| 195 | C | 0.08406 | 2.587 | 0.9721 |
| 930 | A | 0.1748 | 0.362 | 0.9721 |
| 15924 | G | 0.2213 | 0.4246 | 0.9721 |
| 16304 | C | 0.2761 | 0.5116 | 0.9721 |
| 16278 | T | 0.2814 | 0.5042 | 0.9721 |
| 16362 | C | 0.3016 | 0.5364 | 0.9721 |
| 489 | C | 0.3404 | 2.834 | 0.9721 |
| 16192 | T | 0.3474 | 2.803 | 0.9721 |
| 15301 | A | 0.3591 | 0.4302 | 0.9721 |
| 497 | T | 0.3873 | 0.55 | 0.9721 |
| 1719 | A | 0.4289 | 2.367 | 0.9721 |
| 10463 | C | 0.4305 | 0.611 | 0.9721 |
| 16183 | C | 0.4333 | 2.359 | 0.9721 |
| 16189 | C | 0.4369 | 1.59 | 0.9721 |
| 14233 | G | 0.4508 | 0.5917 | 0.9721 |
| 16069 | T | 0.4532 | 2.291 | 0.9721 |
| 15043 | A | 0.4548 | 0.5016 | 0.9721 |
| 9477 | A | 0.457 | 0.6029 | 0.9721 |
| 11719 | G | 0.4662 | 0.7537 | 0.9721 |
| 16519 | T | 0.4688 | 1.368 | 0.9721 |
| 709 | A | 0.4866 | 1.534 | 0.9721 |
| 14766 | C | 0.4974 | 0.7669 | 0.9721 |
| 11812 | G | 0.4985 | 0.625 | 0.9721 |
| 12705 | T | 0.5052 | 1.576 | 0.9721 |
| 1189 | C | 0.5121 | 0.6718 | 0.9721 |
| 16294 | T | 0.5171 | 0.6796 | 0.9721 |
| 16224 | C | 0.6007 | 0.733 | 0.9721 |
| 16311 | C | 0.6214 | 0.7818 | 0.9721 |
| 13708 | A | 0.6334 | 1.48 | 0.9721 |
| 14167 | T | 0.6395 | 0.7586 | 0.9721 |
| 9698 | C | 0.6613 | 0.7722 | 0.9721 |
| 10550 | G | 0.6613 | 0.7722 | 0.9721 |
| 15607 | G | 0.6627 | 0.7483 | 0.9721 |
| 15928 | A | 0.6627 | 0.7483 | 0.9721 |
| 13368 | A | 0.6627 | 0.7483 | 0.9721 |

**Additional file 6: Table S4 (Continued). Association analysis of mtDNA SNPs with acute vs gradual onset in ME/CFS patients.**

| Base-pair | Minor allele | Nominal p-value | Odds Ratio | Benjamini-Hochberg FDR |
| --- | --- | --- | --- | --- |
| 10398 | G | 0.6737 | 0.8233 | 0.9721 |
| 11299 | C | 0.6748 | 0.7807 | 0.9721 |
| 9055 | A | 0.6843 | 0.7876 | 0.9721 |
| 12308 | G | 0.6857 | 1.198 | 0.9721 |
| 12372 | A | 0.6857 | 1.198 | 0.9721 |
| 11467 | G | 0.6954 | 1.191 | 0.9721 |
| 2706 | A | 0.7002 | 0.8618 | 0.9721 |
| 1811 | G | 0.7065 | 1.234 | 0.9721 |
| 3010 | A | 0.7169 | 0.8414 | 0.9721 |
| 73 | A | 0.7185 | 0.8695 | 0.9721 |
| 11914 | A | 0.7204 | 0.7313 | 0.9721 |
| 7028 | C | 0.7263 | 0.8729 | 0.9721 |
| 1888 | A | 0.7318 | 0.7969 | 0.9721 |
| 16126 | C | 0.7427 | 1.204 | 0.9721 |
| 10873 | C | 0.7507 | 0.7592 | 0.9721 |
| 150 | T | 0.7712 | 1.226 | 0.9721 |
| 9540 | C | 0.7777 | 0.7833 | 0.9721 |
| 152 | C | 0.7799 | 1.145 | 0.9721 |
| 8701 | G | 0.7997 | 0.8028 | 0.9721 |
| 4917 | G | 0.8126 | 1.184 | 0.9721 |
| 13617 | C | 0.8287 | 0.852 | 0.9721 |
| 12612 | G | 0.8376 | 1.19 | 0.9721 |
| 11251 | G | 0.841 | 1.121 | 0.9721 |
| 3197 | C | 0.8733 | 0.8875 | 0.9721 |
| 15452 | A | 0.8892 | 1.082 | 0.9721 |
| 16093 | C | 0.9014 | 0.8994 | 0.9721 |
| 16223 | T | 0.9099 | 1.073 | 0.9721 |
| 14905 | A | 0.9103 | 0.9301 | 0.9721 |
| 16270 | T | 0.9165 | 1.092 | 0.9721 |
| 4216 | C | 0.9637 | 0.9759 | 0.9807 |
| 8697 | A | 0.9646 | 1.033 | 0.9807 |
| 14798 | C | 0.9667 | 1.024 | 0.9807 |
| 3480 | G | 0.9812 | 0.9853 | 0.9812 |
