# Supplementary material for: Mitochondrial DNA variants correlate with symptoms in myalgic encephalomyelitis/chronic fatigue syndrome
Source: J Transl Med. 2016 Jan 20;14:19. doi: 10.1186/s12967-016-0771-6 (PMC4719218; doi:10.1186/s12967-016-0771-6)
Supplement: Supplementary file 7 — 10.1186/s12967-016-0771-6 Association analysis of mtDNA SNPs in males. [file 12967_2016_771_MOESM7_ESM.docx]

**Additional file 7: Table S5. Association analysis of mtDNA SNPs in males.**

| Base-pair | Nominal p-value | Benjamini-Hochberg FDR |
| --- | --- | --- |
| 195 | 0.06027 | 0.8574 |
| 152 | 0.07893 | 0.8574 |
| 15043 | 0.08537 | 0.8574 |
| 8251 | 0.1048 | 0.8574 |
| 4529 | 0.1173 | 0.8574 |
| 10238 | 0.1219 | 0.8574 |
| 10034 | 0.1219 | 0.8574 |
| 13780 | 0.1297 | 0.8574 |
| 16362 | 0.1297 | 0.8574 |
| 12501 | 0.1299 | 0.8574 |
| 5147 | 0.1609 | 0.8885 |
| 11914 | 0.1615 | 0.8885 |
| 8697 | 0.1909 | 0.9503 |
| 10463 | 0.2323 | 0.9503 |
| 16162 | 0.2569 | 0.9503 |
| 1719 | 0.2951 | 0.9503 |
| 15924 | 0.3056 | 0.9503 |
| 4917 | 0.3273 | 0.9503 |
| 1888 | 0.3643 | 0.9503 |
| 16296 | 0.41 | 0.9503 |
| 1811 | 0.4451 | 0.9503 |
| 7028 | 0.4825 | 0.9503 |
| 2706 | 0.5085 | 0.9503 |
| 14233 | 0.5098 | 0.9503 |
| 11812 | 0.5102 | 0.9503 |
| 12705 | 0.5213 | 0.9503 |
| 16304 | 0.5425 | 0.9503 |
| 13368 | 0.5425 | 0.9503 |
| 15928 | 0.5425 | 0.9503 |
| 14905 | 0.5425 | 0.9503 |
| 15607 | 0.5425 | 0.9503 |
| 3010 | 0.5628 | 0.9503 |
| 11251 | 0.5644 | 0.9503 |
| 16223 | 0.5645 | 0.9503 |
| 16294 | 0.5684 | 0.9503 |
| 14798 | 0.5684 | 0.9503 |
| 10398 | 0.5785 | 0.9503 |
| 146 | 0.5932 | 0.9503 |
| 709 | 0.5945 | 0.9503 |

**Additional file 7: Table S5 (Continued). Association analysis of mtDNA SNPs in males.**

| Base-pair | Nominal p-value | Benjamini-Hochberg FDR |
| --- | --- | --- |
| 16311 | 0.6339 | 0.9503 |
| 73 | 0.6506 | 0.9503 |
| 12308 | 0.6627 | 0.9503 |
| 12372 | 0.6627 | 0.9503 |
| 16519 | 0.6747 | 0.9503 |
| 16093 | 0.697 | 0.9503 |
| 13617 | 0.697 | 0.9503 |
| 4216 | 0.7007 | 0.9503 |
| 11467 | 0.7027 | 0.9503 |
| 11719 | 0.7055 | 0.9503 |
| 150 | 0.744 | 0.971 |
| 9477 | 0.7503 | 0.971 |
| 15452 | 0.7813 | 0.981 |
| 497 | 0.7878 | 0.981 |
| 16189 | 0.8349 | 1 |
| 9055 | 0.8457 | 1 |
| 14766 | 0.8488 | 1 |
| 3480 | 0.9195 | 1 |
| 10550 | 0.9488 | 1 |
| 9698 | 0.9488 | 1 |
| 16224 | 0.9731 | 1 |
| 11299 | 0.9741 | 1 |
| 930 | 0.9775 | 1 |
| 1189 | 0.9775 | 1 |
| 16270 | 0.9816 | 1 |
| 14167 | 1 | 1 |
| 16126 | 1 | 1 |
