# Supplementary material for: Mitochondrial DNA variants correlate with symptoms in myalgic encephalomyelitis/chronic fatigue syndrome
Source: J Transl Med. 2016 Jan 20;14:19. doi: 10.1186/s12967-016-0771-6 (PMC4719218; doi:10.1186/s12967-016-0771-6)
Supplement: Supplementary file 8 — 10.1186/s12967-016-0771-6 Association analysis of mtDNA SNPs in females. [file 12967_2016_771_MOESM8_ESM.docx]

**Additional file 8: Table S6. Association analysis of mtDNA SNPs in females.**

| Base-pair | Nominal p-value | Benjamini-Hochberg FDR |
| --- | --- | --- |
| 709 | 0.006803 | 0.3131 |
| 11719 | 0.02359 | 0.3131 |
| 8697 | 0.02834 | 0.3131 |
| 14766 | 0.04358 | 0.3131 |
| 9540 | 0.04606 | 0.3131 |
| 10463 | 0.04836 | 0.3131 |
| 13368 | 0.05082 | 0.3131 |
| 15607 | 0.05082 | 0.3131 |
| 15928 | 0.05082 | 0.3131 |
| 146 | 0.05092 | 0.3131 |
| 16294 | 0.05177 | 0.3131 |
| 10873 | 0.05549 | 0.3131 |
| 8701 | 0.05733 | 0.3131 |
| 1888 | 0.06875 | 0.3472 |
| 16189 | 0.07335 | 0.3472 |
| 4917 | 0.08097 | 0.3593 |
| 4216 | 0.08846 | 0.3695 |
| 14798 | 0.0954 | 0.372 |
| 73 | 0.09954 | 0.372 |
| 11914 | 0.105 | 0.3729 |
| 15301 | 0.1128 | 0.3813 |
| 3480 | 0.1218 | 0.3919 |
| 14905 | 0.1269 | 0.3919 |
| 12705 | 0.1338 | 0.3959 |
| 11251 | 0.1414 | 0.4017 |
| 1189 | 0.1502 | 0.4103 |
| 11812 | 0.1737 | 0.412 |
| 14233 | 0.1737 | 0.412 |
| 16126 | 0.1741 | 0.412 |
| 15452 | 0.1741 | 0.412 |
| 152 | 0.1905 | 0.4364 |
| 16311 | 0.2074 | 0.4602 |
| 10550 | 0.2486 | 0.5184 |
| 11299 | 0.2486 | 0.5184 |
| 1719 | 0.2555 | 0.5184 |
| 2706 | 0.287 | 0.566 |
| 14167 | 0.3032 | 0.572 |
| 16224 | 0.3109 | 0.572 |
| 16223 | 0.3162 | 0.572 |
| 16093 | 0.3305 | 0.572 |

**Additional file 8: Table S6 (Continued). Association analysis of mtDNA SNPs in females.**

| Base-pair | Nominal p-value | Benjamini-Hochberg FDR |
| --- | --- | --- |
| 16270 | 0.3402 | 0.572 |
| 16183 | 0.349 | 0.572 |
| 9698 | 0.3545 | 0.572 |
| 9055 | 0.3545 | 0.572 |
| 7028 | 0.3708 | 0.5772 |
| 195 | 0.374 | 0.5772 |
| 1811 | 0.4105 | 0.6201 |
| 5460 | 0.4366 | 0.6431 |
| 13617 | 0.4477 | 0.6431 |
| 3010 | 0.4607 | 0.6431 |
| 3197 | 0.462 | 0.6431 |
| 15043 | 0.49 | 0.6691 |
| 16298 | 0.5266 | 0.7054 |
| 489 | 0.5424 | 0.7131 |
| 12612 | 0.5589 | 0.7133 |
| 9477 | 0.5626 | 0.7133 |
| 16278 | 0.5762 | 0.7177 |
| 497 | 0.5965 | 0.7301 |
| 930 | 0.6234 | 0.7501 |
| 16192 | 0.6845 | 0.81 |
| 16069 | 0.7093 | 0.8143 |
| 16304 | 0.7246 | 0.8143 |
| 16362 | 0.7292 | 0.8143 |
| 13708 | 0.7425 | 0.8143 |
| 16519 | 0.7455 | 0.8143 |
| 10398 | 0.8797 | 0.9251 |
| 12372 | 0.899 | 0.9251 |
| 12308 | 0.899 | 0.9251 |
| 11467 | 0.899 | 0.9251 |
| 15924 | 0.9179 | 0.931 |
| 150 | 0.9603 | 0.9603 |
