# Supplementary material for: Mitochondrial DNA variants correlate with symptoms in myalgic encephalomyelitis/chronic fatigue syndrome
Source: J Transl Med. 2016 Jan 20;14:19. doi: 10.1186/s12967-016-0771-6 (PMC4719218; doi:10.1186/s12967-016-0771-6)
Supplement: Supplementary file 9 — 10.1186/s12967-016-0771-6 Quantile–quantile plots of symptoms significantly associated with mtDNA SNPs. [file 12967_2016_771_MOESM9_ESM.docx]

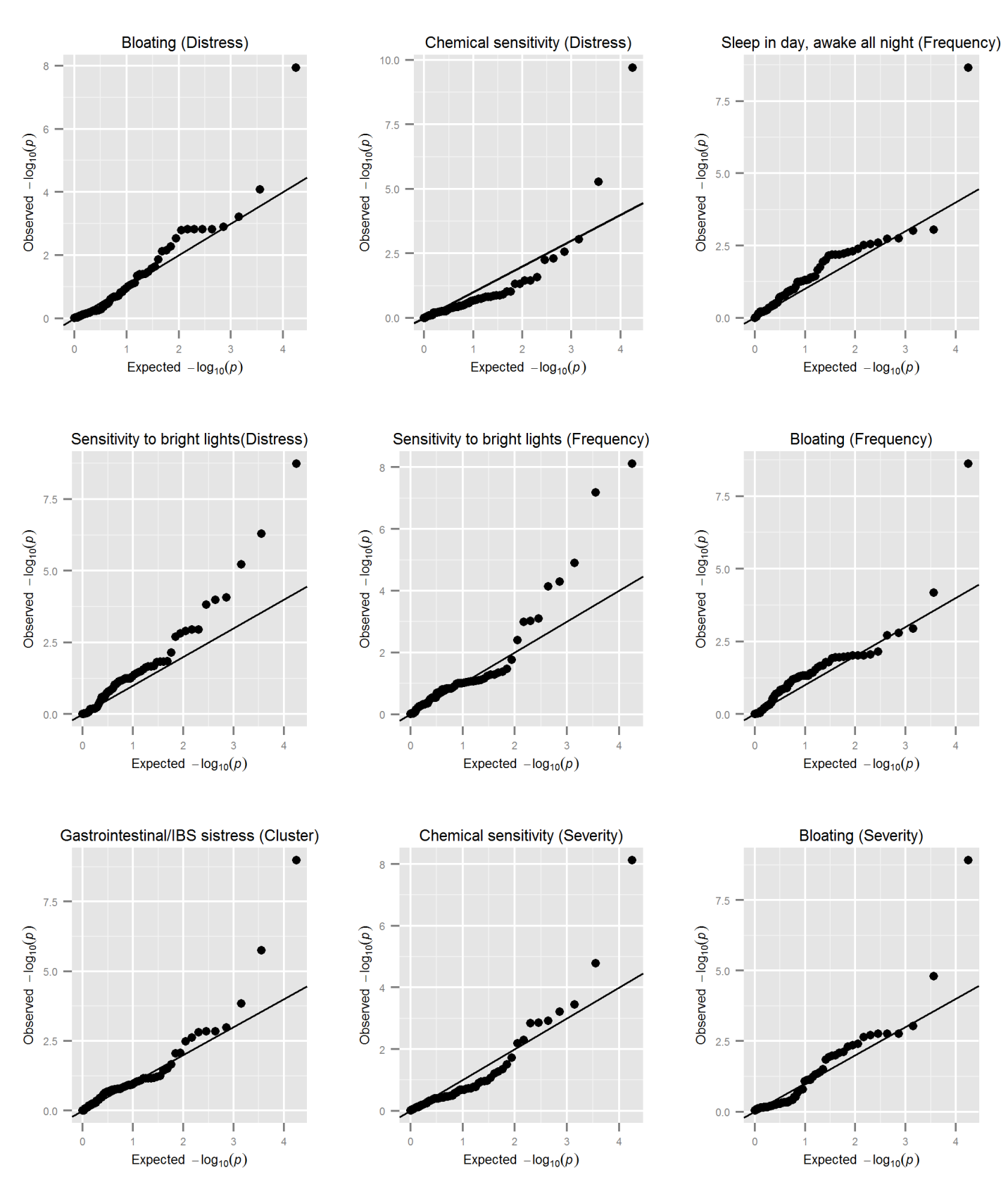


Additional file 9: Fig. S3. Quantile-quantile plots of symptoms significantly associated with mtDNA SNPs. On the x-axis are values representing a uniform distribution of p-values expected under the null hypothesis of no association, while observed values are on the Y. Each point on the graphs represents a different mtDNA SNP. 70 mtDNA SNPs were tested for association with each symptom. Points that fall above the line are more significant than expected under the null hypothesis.
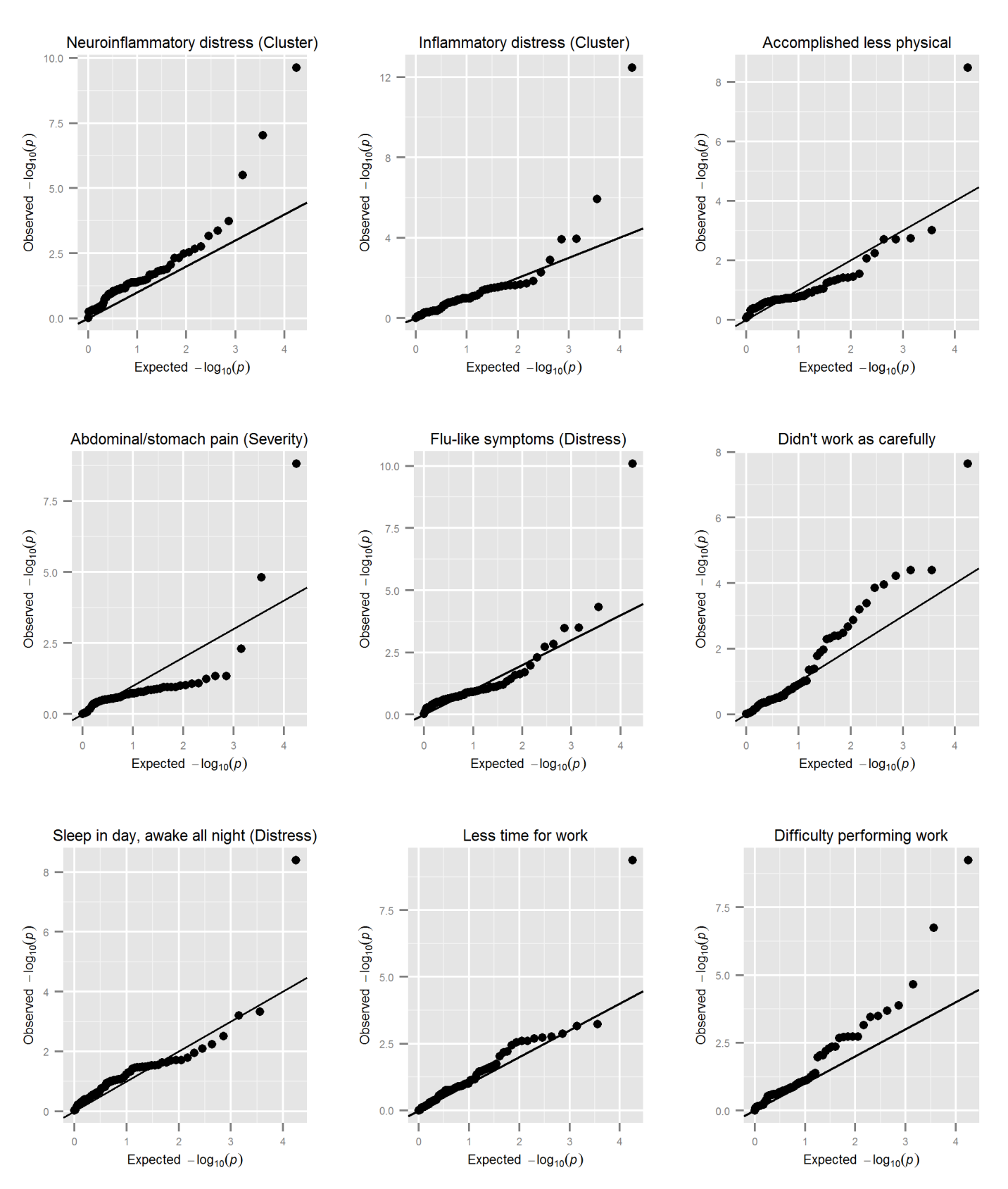


Additional file 9: Fig. S3 (Continued). Quantile-quantile plots of symptoms significantly associated with mtDNA SNPs.


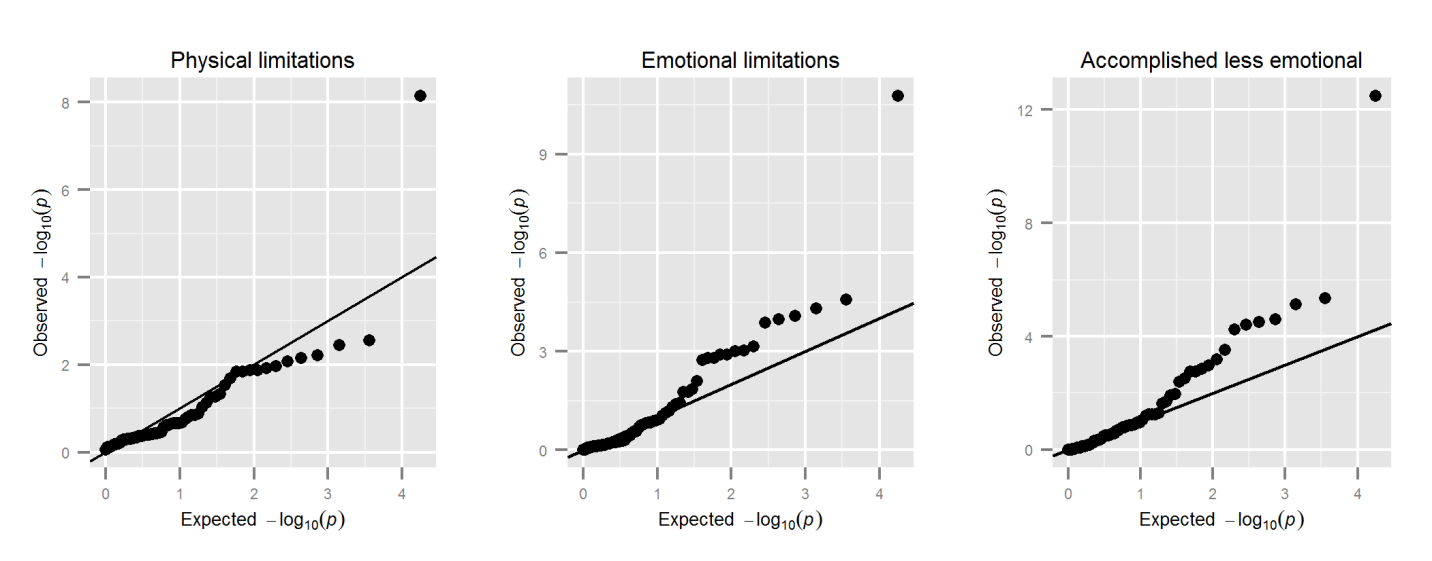


Additional file 9: Fig. S3 (Continued). Quantile-quantile plots of symptoms significantly associated with mtDNA SNPs.
