# Supplementary material for: Mitochondrial DNA variants correlate with symptoms in myalgic encephalomyelitis/chronic fatigue syndrome
Source: J Transl Med. 2016 Jan 20;14:19. doi: 10.1186/s12967-016-0771-6 (PMC4719218; doi:10.1186/s12967-016-0771-6)
Supplement: Supplementary file 10 — 10.1186/s12967-016-0771-6 Boxplots of SF-36/DSQ symptom scores associated with mtDNA alleles. [file 12967_2016_771_MOESM10_ESM.docx]

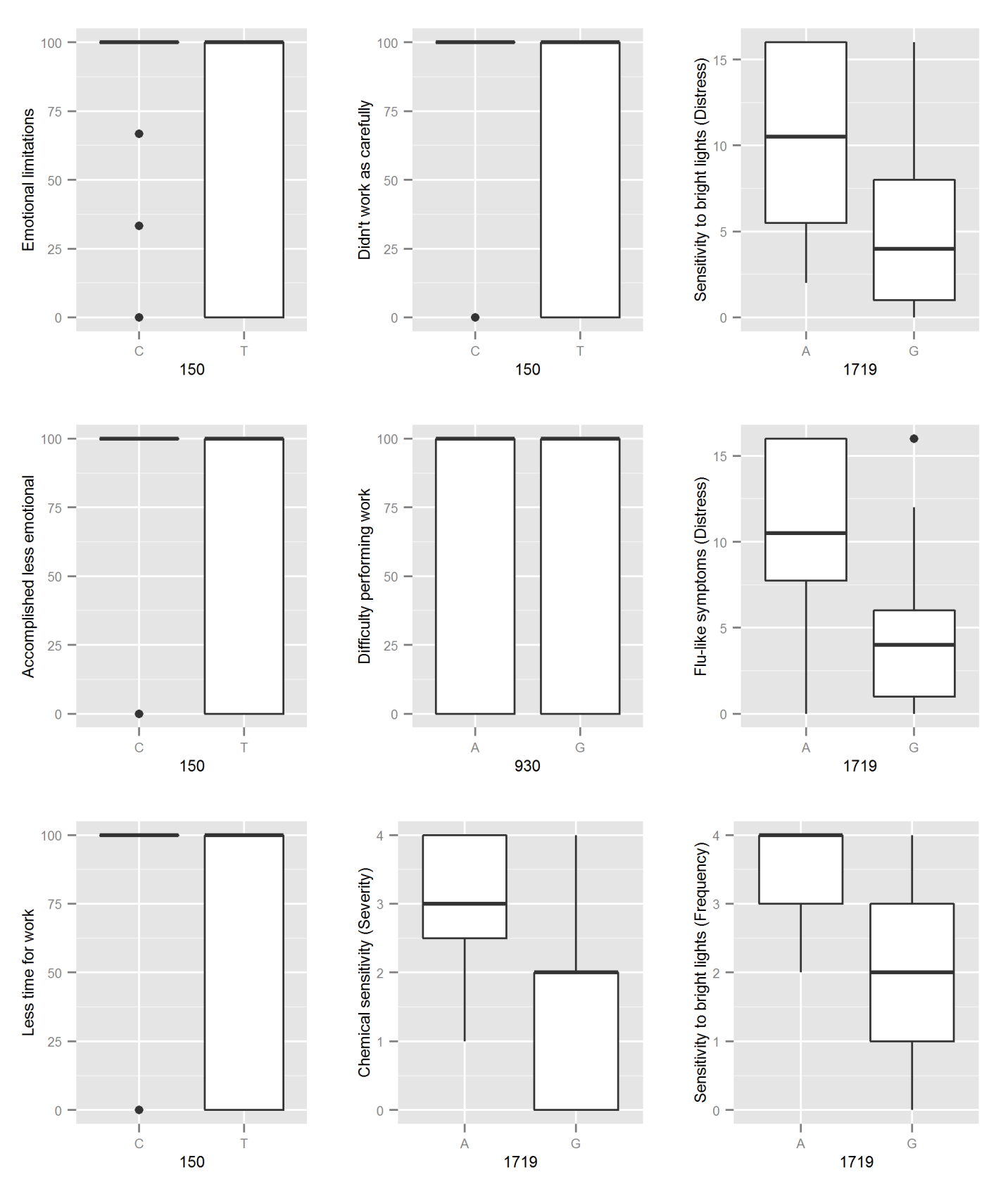


**Additional file 10: Fig. S4. Boxplots of SF-36/DSQ symptom scores associated with mtDNA alleles.** Boxplots of 18 significant associations not shown in Figure 1. The x-axis shows mtDNA position and alleles while the y-axis shows symptom scores and is labelled with descriptions. Some symptom descriptions were shortened for this figure. Chemical sensitivity refers to “Some smells, foods, medications, or chemicals make you feel sick”. Subjects indicated whether they accomplished less due to emotional factors or because of physical limitations.
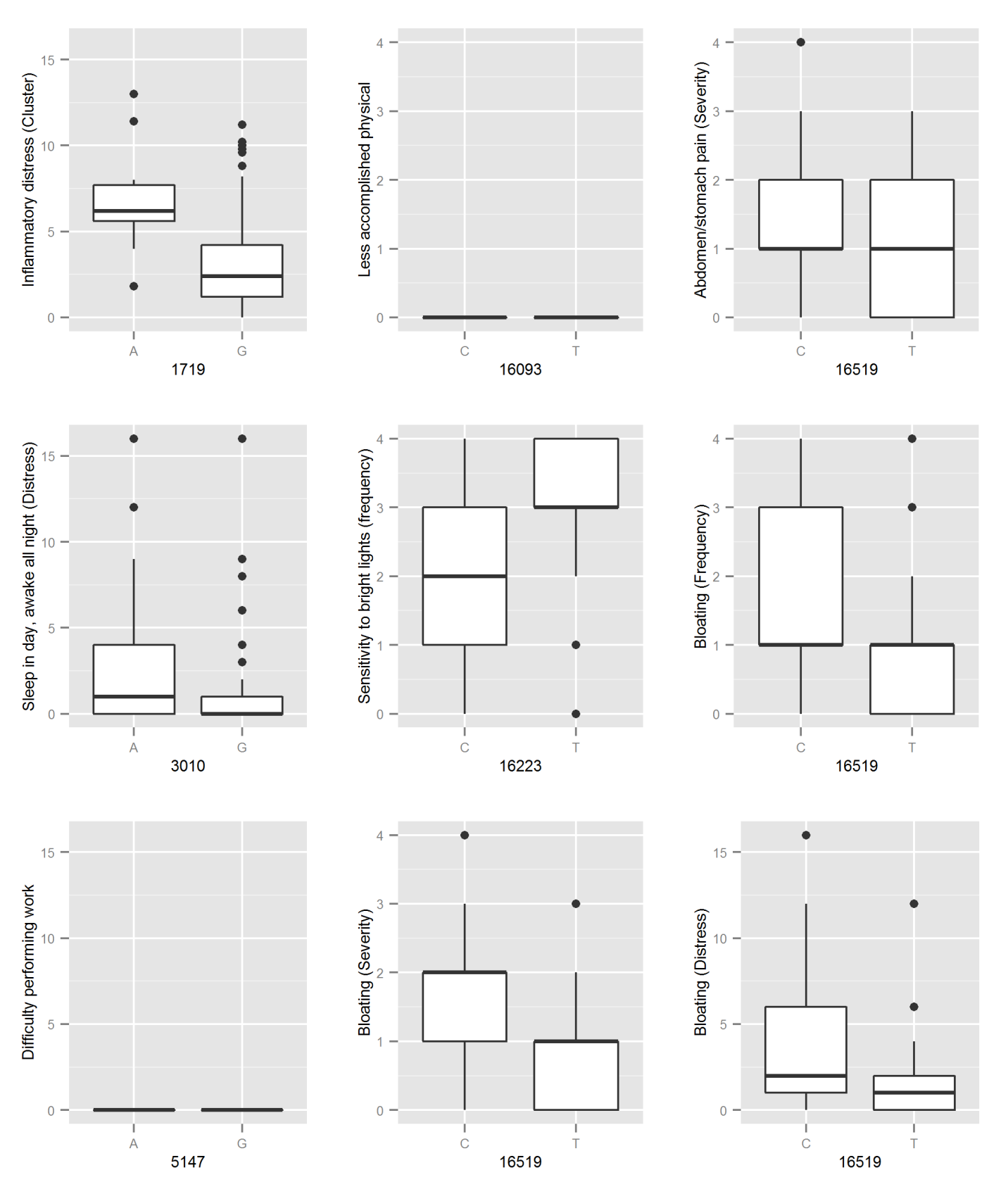


Additional file 10: Fig. S4 (Continued). Boxplots of SF-36/DSQ symptom scores associated with mtDNA alleles.
