# Supplementary material for: Mitochondrial DNA variants correlate with symptoms in myalgic encephalomyelitis/chronic fatigue syndrome
Source: J Transl Med. 2016 Jan 20;14:19. doi: 10.1186/s12967-016-0771-6 (PMC4719218; doi:10.1186/s12967-016-0771-6)
Supplement: Supplementary file 11 — 10.1186/s12967-016-0771-6 Comparison of HPUI distribution between individuals with acute and gradual CFS onset. [file 12967_2016_771_MOESM11_ESM.docx]

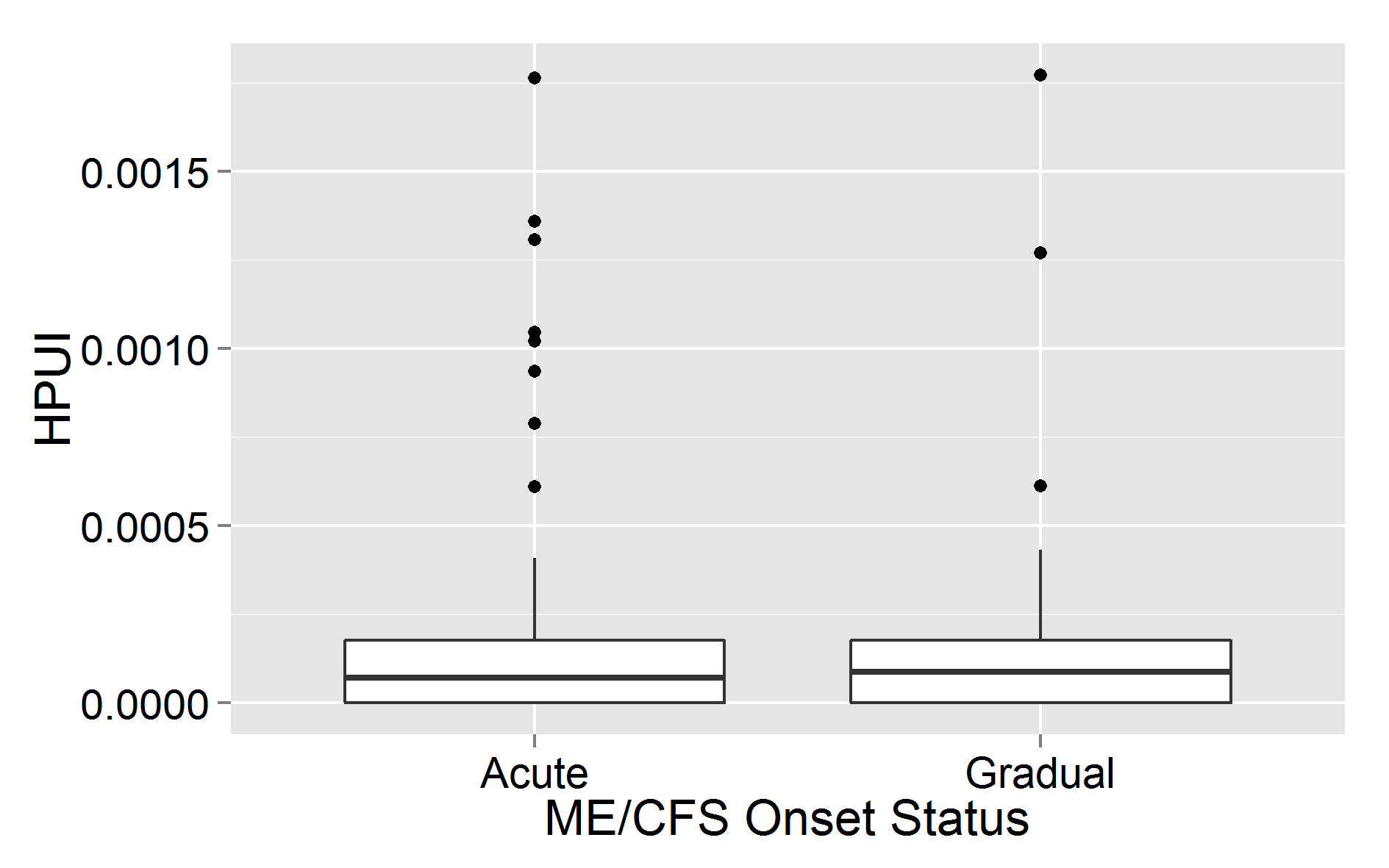


Additional file 11: Fig. S5. Comparison of HPUI distribution between individuals with acute and gradual CFS onset.
