# Supplementary material for: Mitochondrial DNA variants correlate with symptoms in myalgic encephalomyelitis/chronic fatigue syndrome
Source: J Transl Med. 2016 Jan 20;14:19. doi: 10.1186/s12967-016-0771-6 (PMC4719218; doi:10.1186/s12967-016-0771-6)
Supplement: Supplementary file 12 — 10.1186/s12967-016-0771-6 Comparison of HPUI distribution between individuals who have experienced ME/CFS for less than 3 years and more than 3 years. [file 12967_2016_771_MOESM12_ESM.docx]

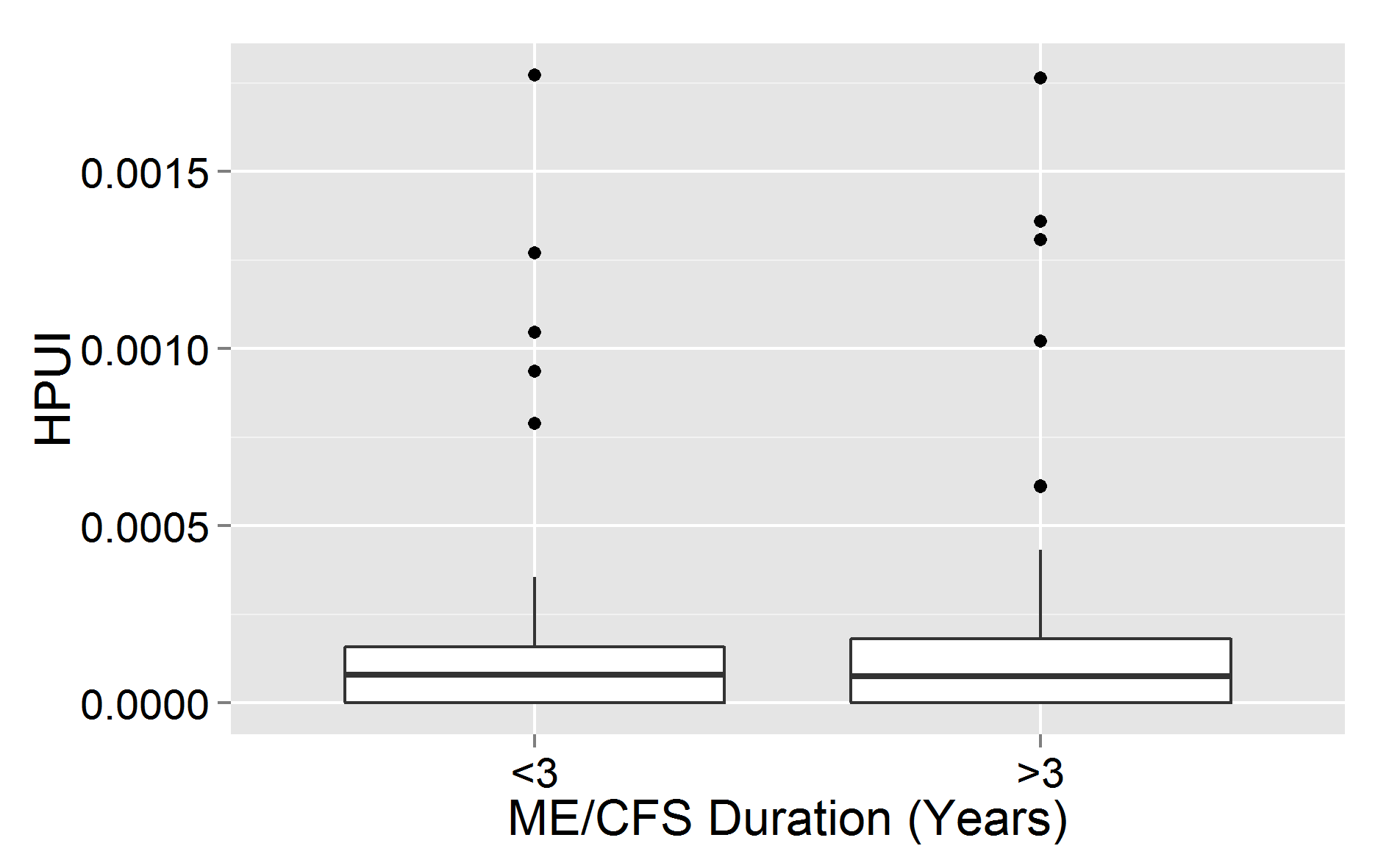


Additional file 12: Fig. S6. Comparison of HPUI distribution between individuals who have experienced ME/CFS for less than 3 years and more than 3 years.
